# A novel framework for elucidating the effect of mechanical loading on the geometry of ovariectomized mouse tibiae using principal component analysis

**DOI:** 10.3389/fbioe.2024.1469272

**Published:** 2024-10-22

**Authors:** Stamatina Moraiti, Vee San Cheong, Enrico Dall’Ara, Visakan Kadirkamanathan, Pinaki Bhattacharya

**Affiliations:** ^1^ Department of Mechanical Engineering, University of Sheffield, Sheffield, United Kingdom; ^2^ INSIGNEO Institute for in silico Medicine, University of Sheffield, Sheffield, United Kingdom; ^3^ Future Health Technologies Programme, Singapore-ETH Centre, Create campus, Singapore, Singapore; ^4^ School of Medicine and Population Health, University of Sheffield, Sheffield, United Kingdom; ^5^ Department of Automatic Control and Systems Engineering, University of Sheffield, Sheffield, United Kingdom

**Keywords:** mouse tibia, osteoporosis, mechanical loading, bone morphometry, principal component analysis (PCA)

## Abstract

**Introduction:**

Murine models are used to test the effect of anti-osteoporosis treatments as they replicate some of the bone phenotypes observed in osteoporotic (OP) patients. The effect of disease and treatment is typically described as changes in bone geometry and microstructure over time. Conventional assessment of geometric changes relies on morphometric scalar parameters. However, being correlated with each other, these parameters do not describe separate fractions of variations and offer only a moderate insight into temporal changes.

**Methods:**

The current study proposes a novel image-based framework that employs deformable image registration on *in vivo* longitudinal images of bones and Principal Component Analysis (PCA) for improved quantification of geometric effects of OP treatments. This PCA-based model and a novel post-processing of score changes provide orthogonal modes of shape variations temporally induced by a course of treatment (specifically *in vivo* mechanical loading).

**Results and Discussion:**

Errors associated with the proposed framework are rigorously quantified and it is shown that the accuracy of deformable image registration in capturing the bone shapes (∼1 voxel = 10.4 μm) is of the same order of magnitude as the relevant state-of-the-art evaluation studies. Applying the framework to longitudinal image data from the midshaft section of ovariectomized mouse tibia, two mutually orthogonal mode shapes are reliably identified to be an effect of treatment. The mode shapes captured changes of the tibia geometry due to the treatment at the anterior crest (maximum of 0.103 mm) and across the tibia midshaft section and the posterior (0.030 mm) and medial (0.024 mm) aspects. These changes agree with those reported previously but are now described in a compact fashion, as a vector field of displacements on the bone surface. The proposed framework enables a more detailed investigation of the effect of disease and treatment on bones in preclinical studies and boosts the precision of such assessments.

## 1 Introduction

Osteoporosis is one of the most severe and common skeletal diseases that reduces bone mineral density (BMD), and diminishes bone quality and structural integrity, leading to weaker bones and a higher risk of fracture. Treatment strategies such as exercise aim to enhance bone structure by stimulating new bone tissue formation and reducing bone loss (Rodan and Martin, 2000). Research in this area focuses on elaborating how bones respond to external mechanical stimuli. Murine models play a crucial role in investigating treatment strategies for osteoporosis due to their rapid response to interventions (Jilka, 2013). In particular, the ovariectomized murine model is an accepted model of estrogen deficiency that accelerates bone resorption as observed in post-menopausal osteoporotic patients (Bouxsein et al., 2005). *In vivo* micro–Computed Tomography (microCT) enables the longitudinal acquisition of high-resolution images of peripheral bones (e.g., the tibia, caudal vertebrae) in mice (Bouxsein et al., 2010). For example, this approach has been used to study the effect of aging, ovariectomy, mechanical loading and pharmacological interventions (Akhter and Recker, 2021; Dall’Ara et al., 2016; Levchuk et al., 2014; Viceconti and Dall’Ara, 2019).

Established imaging and image processing protocols for *in vivo* microCT images of murine bones enable the quantification of spatio-temporal changes in geometry, microstructure and bone adaptation over time (Birkhold et al., 2015; Javaheri et al., 2020; Jepsen et al., 2015; Roberts et al., 2019; Oliviero et al., 2019; van ’t Hof and Dall’Ara, 2019; Gasser et al., 2005). Mouse bone geometric variations are often reported as variations of scalar geometric properties obtained from standard morphometric analysis of 3D images (Bouxsein et al., 2010). Such geometric properties, for, e.g., cortical thickness, area, volume, eccentricity, and moments of inertia, allow comparisons with older similar histomorphometric measures (Dempster et al., 2013; Iida-Klein et al., 2007; Zhou et al., 2003). As histomorphometry analysis involves 2D *ex vivo* bone samples using microscopy or structural analysis in *ex vivo* images (Miller et al., 2021; Rooney et al., 2023; Monzem et al., 2023; Sugiyama et al., 2008; De Souza et al., 2005; Weatherholt et al., 2013), standard morphometric analysis is also focused on short, pseudo-2D regions, such as the tibia midshaft, even when applied to 3D *in vivo* images (Roberts et al., 2019; Holguin et al., 2014; Silva et al., 2012). As such, scalar geometric assessment is based on assumptions and simplifications of the geometry and produces measures that are averaged over the examined section. Despite the advancement of *in vivo* longitudinal imaging, the methodology to analyze such images for a complete assessment of bone shape changes remains underdeveloped.

A limitation of the state-of-the-art approach is that it provides averaged scalar quantifiers to characterize the structure which effectively ignores the 3D complexity of bone shape changes. However, other preclinical studies provide evidence of localized bone geometry changes for, e.g., due to external mechanical loading. Miller et al. (2021) measured the local thickness on four cross sections (25%, 37%, 50% and 75% of length) of murine tibiae and showed that the local changes due to mechanical loading depended on the location and the loading magnitude. Zhang et al. (2019) introduced a novel wavelet transform framework and found that over an 8-week period, changes observed in mouse tibia geometry were heterogeneous and contained both low- and high-frequency components in space. Javaheri et al. (2020) used superimposition and rigid registration measures and showed that the mouse tibia adapted to a short-term loading regime, but after the regime concluded, these adaptations were only partially retained. Other computational studies predict local bone adaptation that also cannot be described by standard morphometric parameters alone. For example, Cheong et al. (2020a) suggested that mechanical loading of mouse tibia predominantly impacts the periosteum. Carriero et al. (2018) combined 3D fluorochrome mapping and finite element modelling and showed prominent endosteal formation in the lateral aspect of the mouse tibia and decreased turnover distal to the midshaft. Although the aforementioned techniques have revealed new insights regarding local bone adaptation, it remains unclear which geometrical features explain the most variability within a population and which consistently change due to disease or treatment.

Another drawback of the scalar morphometric properties is their mutual interdependence, i.e., non-negligible covariance. One example is the correlation between cortical thickness and cortical area, with the latter being described as a function of the former. This explains why studies report concurrent changes over time in both parameters (Roberts et al., 2020). Therefore, not only do the variations in individual standard morphometric parameters overlap with each other, but also, when combined, these also only partially explain the total variation in bone geometry. This also points to a potential challenge in using the effect sizes of the standard morphometric parameters (changes in bone shapes), when this assessment aims to represent the efficacy of a candidate treatment on bone geometry.

A potential alternative to the currently incomplete assessment of geometric changes is to use PCA to extract ‘patterns’ of bone geometry variations. This is promising because each ‘pattern’ (or PC mode) encodes local variations throughout the bone surface and is guaranteed to explain an independent fraction of the total bone geometry variation. More details on methodology and application of PCA in shape modelling can be found in monographs such as Davies et al. (2008).

PCA has been used in various applications. One common use in bone research is to identify shape and/or intensity variations on osteoporotic bones to improve osteoporosis care. The literature reviews of Castro-Mateos et al. (2014) and Grassi et al. (2021) detail the major methodological characteristics of the PCA models. Their various applications include segmentation, preoperative planning, 2D-to-3D intensity and shape reconstruction using 2D clinical images (Dual-energy X-ray Absorptiometry, DXA), Finite Element Modelling and investigation of bone fracture. More specifically, in the context of the human femur, PCA has been used to predict hip fracture risk combining shape and intensity modes derived by DXA (Aldieri et al., 2020). Additionally, it has been used to reduce the dependence on expensive imaging modalities (e.g., CT/MRI) in pre-operative planning (Rajamani et al., 2007; Barratt et al., 2008). Moreover, it was developed to generate statistical models of geometry and material properties of the human femur (Bryan et al., 2010). In mouse bone research, the use of PCA is relatively limited. Killian et al. (2019) used PCA to investigate the deformity of dysplastic murine hips and Chan et al. (2012) used PCA to identify the major distal femoral geometrical features that temporally vary during bone maturation. Both these studies focused on sections of bones. A larger scale model developed by Brown et al. (2017) investigated abnormalities in the murine hind paws with rheumatoid arthritis.

Assessing shape variations requires image processing to enable direct comparison between the shape observations (Castro-Mateos et al., 2014). Chan et al. (2012) performed an automatic landmarking approach for murine bones and obtained an atlas with 412 anatomical landmarks. This bone structure was a segment of the distal femur extracted from *ex vivo* images of mice at different ages (resolution 9 μm). Since the images were acquired in a cross-sectional experimental design, captured variations due to bone maturation could not be classed into temporal changes. Therefore, they were reported as variations from the mean shape. Additionally, the performed rigid registration protocol involved isotropic scaling and eliminated the size variations related to length and area. Killian et al. (2019) also focused on subsections of bones in the hip joint, scanned using *in vivo* micro–Computed Tomography (resolution 21 μm). In that study, the surfaces were scaled up and an automatic landmarking algorithm in ShapeWorks (Cates et al., 2017) was used to discretize the shapes using 2048 points. Although a comparison between disease severity also appears feasible using this approach, Killian et al. (2019) examined only one disease stage (severe) in their study. Brown et al. (2017) applied an automated method for discretizing and registering the meshes (∼200,000 vertices) from volumetric microCT images of the mouse hind paw (resolution ∼14 μm). Finally, Hoshino et al. (2023) analyzed murine skull variations to investigate dysmorphology (resolution ∼62 μm), using 33 landmarks to define the skull shape.

The findings from the literature suggest that instance alignment, shape correspondence and reliability analysis emerge as three important aspects common to all methodologies for conducting a Statistical Shape Models (SSM). The protocol of instance alignment varies among different studies. This step starts with defining an atlas (alternatively called template or reference) that can be either the mean shape or a random choice of one shape observation. Then the shapes are aligned to the reference. This alignment is either applied to the images, using rigid body movements, or applied to meshes, using algorithms based on distance metrics. Isotropic scaling of shapes is also sometimes used in the literature to disregard size variations. The number of landmarks used to achieve correspondence among shapes, varies depending on factors such as examined structure, image resolution, discretization and mapping method. Correspondence is primarily accomplished via deformable registration using B-Spline interpolation or other types of interpolation such as Kernel-based in Deformetrica (Bône et al., 2018; Fornai et al., 2021; Heutinck et al., 2021) Past studies highlight that the choices made in each step depend on the goal of each study, and the reliability of each step can influence the reliability of any shape model derived from them. For example, a small number of landmarks may lead to unrepresentative shape descriptions, or missing morphologic information of the examined structure, resulting in a lack of robustness to the shape analysis.

The objective of the present study is to develop a robust and accurate framework that can be used to assess the effects of disease (here, ovariectomy-induced bone loss) and treatment (here, passive mechanical loading) on the 3D geometry of long bones (here, tibial midshaft section) in a murine population (here, female adult C57BL/6 mice). This novel framework combines *in vivo* longitudinal imaging, deformable registration, PCA and statistical analysis for treatment investigation in preclinical studies.

## 2 Materials and methods

**FIGURE 1:**
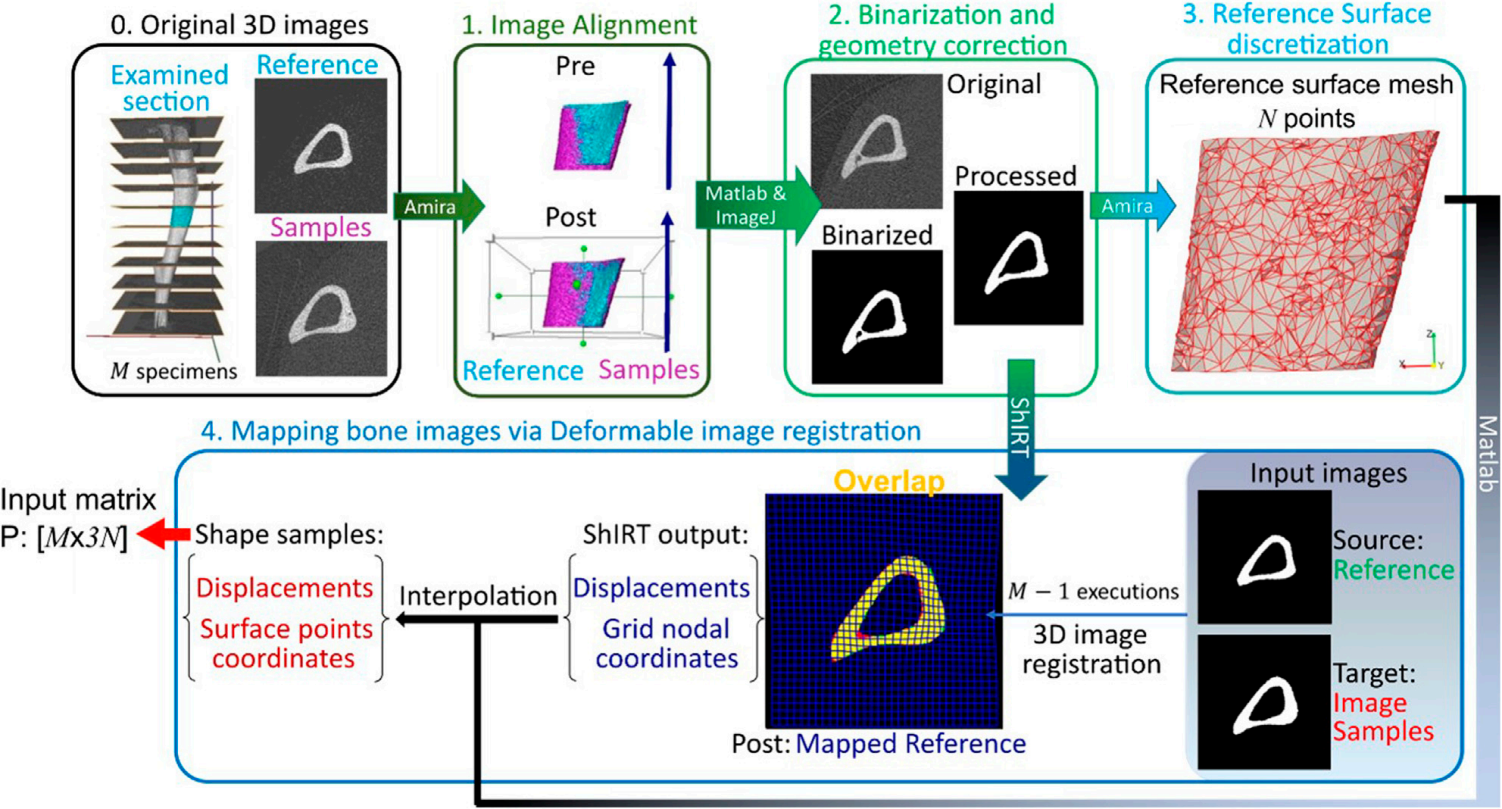
Framework flowchart. Step 0: The grayscale image slices corresponding to the midshaft section are extracted for each of the *M* images of mouse tibia. Step1: All images are aligned to a reference image sample previously registered to its anatomical coordinate system. Step 2: The greyscale images are binarized and corrected to enforce topological equivalence. Step 3: The reference surface mesh, consisting of *N* nodes with *x*, *y* and *z* coordinates, is extracted from the binarized reference image. Step 4: The reference bone is mapped to all other image samples by applying a deformable image registration algorithm using the Sheffield Image Registration Toolkit (ShIRT). The deformation field is applied to the reference surface mesh leading to individual surface meshes for each bone shape observation. The periosteum and endosteum surface mesh for each mouse image constitutes the rows of the PCA input **P**.

The framework developed in this study is used to analyze image data obtained from past animal experiments. Sections 2.1, 2.2 recall details from specific past studies concerning the mice used, the interventions performed, and longitudinal microCT images acquired following a well-established imaging protocol (Lu et al., 2016). The details of the new framework follow from section 2.3 onwards. Sections 2.3–2.6 describe how the existing images are processed to extract bone geometry information. A validated and robust deformable registration technique named Sheffield Image Registration Toolkit (ShIRT) (Barber and Hose, 2005) is used here for the first time to map bones within a population of mice over a course of treatment. The mapping leads to differences in coordinates of “anatomically similar” locations on the endosteal and periosteal surfaces (see also schematic in Figure 1). In section 2.7, these differences in bone surface are decomposed into mutually orthogonal mode shapes using PCA, and section 2.8 describes a novel post-processing statistical analysis of PCA scores that is used to identify effects on geometry due to disease and treatment.

### 2.1 Animals and interventions

The experimental data were taken from two previous murine studies published elsewhere (Roberts et al., 2019; 2020). Therein, all experimental procedures were performed under the British Home Office licenses (PPL 40/3499 and PF61050A3), complied with the UK Animals (Scientific Procedures) Act 1986 and were ethically approved by the local Research Ethics Committee of the University of Sheffield. Female virgin C57BL/6 mice (*n* = 5, Roberts et al. (2019); *n* = 6; Roberts et al. (2020)) were housed starting at 13 weeks old. All mice were skeletally mature at the time of purchase. The housing conditions were temperature of 22°C, with a 12-h dark/light cycle, and *ad libitum* access to protein rodent diet and water.

All mice were ovariectomized at 14 weeks old to generate estrogen deficiency (Turner, 2001). The five mice comprising the control group “OVX” (Roberts et al., 2019) remained untreated throughout the study. The six mice in the treatment group “OVX + ML” (Roberts et al., 2020) were subjected to mechanical loading using the tibia loading model (Nepal et al., 2023) 3 days per week at 19 and 21 weeks old. The right tibia was uniaxially compressed along the superior–inferior axis. Waveform load cycles were performed, with a peak of 12 N (held for 0.2 s) and a 10 s interval between each cycle. All mice were humanely killed at 24 weeks old.

### 2.2 *In vivo* imaging and image alignment

The right tibia of each mouse was scanned using *in vivo* micro–Computed Tomography (microCT, VivaCT80, Scanco Medical, Switzerland) every 2 weeks between weeks 14 and 24 (Roberts et al., 2019; 2020). The scanning parameters were: 10.4 μm isotropic voxel size, voltage of 55 keV, intensity of 145 μA, field of view of 32 mm, 1500/750 samples/projections, integration time 100 m. The microCT images of all mice and time-points were co-registered as follows (Cheong et al., 2020b; Cheong et al., 2021). First, the growth plate, the fibula and the condyles were removed from all microCT images. The proximal and distal cropping resulted in examining 80% of the total length. Next, the 3D image of one random tibia at 14 weeks was considered as a reference. The *z*-axis was aligned to the reference tibia anatomical axial direction and the *x*-axis was aligned to the anterior–posterior direction, such that the *x*–*z* plane bisected the midpoint of the line joining the centers of the articular surfaces of the medial and lateral condyles (Lu et al., 2016). The remaining images were rigidly registered to the reference tibia image using Normalized Mutual Information as the similarity metric and Lanczos interpolation (Amira, v5.4.3, FEI Visualization Sciences Group, France).

In the present study, the registered images of each mouse from two time-points (18 and 24 weeks) extracted from Cheong et al. (2020b) were analyzed. Note that all mice at 18 weeks present evidence of ovariectomy untreated for a period of 4 weeks. The bones in the “OVX” group at 24 weeks present evidence of ovariectomy untreated for a period of 10 weeks. Lastly, the bones in the “OVX + ML” group at 24 weeks present evidence of ovariectomy untreated for a period of 4 weeks followed by mechanical loading treatment at weeks 19 and 21.

### 2.3 Midshaft section and further alignment

The 80% of the total length was divided into 10 equal longitudinal sections and slices corresponding to a midshaft section (Figure 1, Step 0) measuring 8% of the tibia length were considered. To suppress differences in relative position due to differences in whole tibia length, the cropped tibial midshaft images were rigidly registered to a randomly selected reference cropped image (Figure 1, Step 1). Rigid registration was performed in Amira (v6.3, Thermo Fisher) using Mutual Normalized Information as a similarity metric and Lanczos interpolation.

### 2.4 Binarization and geometry correction

**FIGURE 2:**
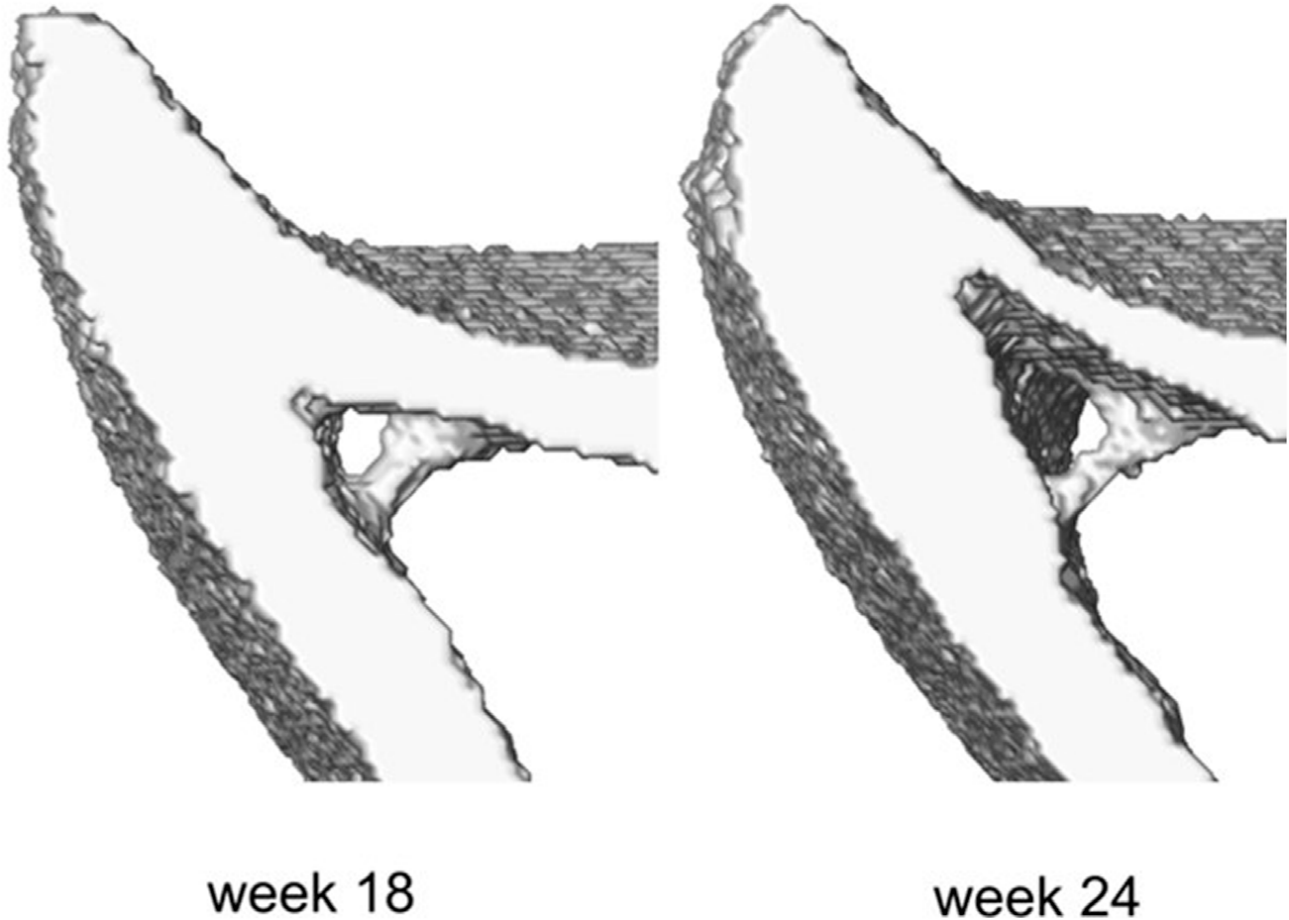
Example of the trabecula feature and its structural evolution over time between weeks 18 and 24 of age.

The midshaft sections typically contain cortical pores and trabeculae. These isolated features do not occur at anatomically similar locations across bones, and as such their presence poses challenges in mapping the periosteal and endosteal surfaces as desired. To suppress these features, the following semi-automatic process was used (Figure 1, Step 2). Firstly, every image was binarized using a single-level threshold. The arithmetic means of the peaks in the intensity histogram corresponding to the background and bone voxels was used as the threshold value. Then all 2D image slices were individually considered and holes with perimeter smaller than 50 pixels were automatically identified. The holes were then artificially ‘filled’ by applying simultaneous dilation and erosion algorithms (Matlab v2021, functions: ‘imdilate’, ‘imerode’). The remaining big hole-like features such as the cavities formed between the endosteum and the trabecula as in Figure 2, were treated manually. The image data at both ages for that mouse were considered, and the number of adjacent slices containing the feature was counted in these images. Since the structural evolution of trabeculae has a direct impact on thickness (Figure 2), if both counts were smaller than seven slices (∼70 μm), then the trabecular feature bounding the hole was identified and deleted from all slices in both images. The remaining hole-like features were classified as either being bound by trabeculae or being cortical pores, looking at their structural changes between the two time points. Features that were not holes, but appeared as notches and transverse gaps in the cortex were classified as early-stage formed trabeculae and transverse cortical pores respectively. After classification, trabeculae were deleted, and cortical pores were filled, leading finally to binarized images of the registered section with well-defined endosteal and periosteal surfaces.

This geometry correction step was evaluated to demonstrate the bone geometry and mechanical alterations due to the simplification of the bone sections. The sensitivity study is elaborated in Supplementary Material 1. Briefly, micro–Finite Element analyses were conducted on the original and processed bone sections. The numbers of common and different elements were assessed to indicate the fraction of the geometric differences between the two types of models. The differences in the strain distribution across the entire bone section and locally around the cortical pores and trabeculae and in the highly deformed areas were also assessed. It was found that the removed features occupied an average of 0.40% of the total number of elements across all mouse samples and the strain distribution of the processed section is not different from the ones of the original bones.

### 2.5 Reference surface discretization

The endosteal and periosteal surfaces bounding the bone volume in the binarized reference image were discretized (Figure 1, Step 3). The discretization was produced using the default settings in Amira v6.3 and is denoted as Mesh-0. It contained 166,639 triangular faces with a mean edge length of 13.9 μm. A coarser discretization of the reference bone surface can potentially reduce the computational demand in the trilinear interpolation and PCA steps of the framework described later. Therefore, the above mesh was coarsened successively using Amira v6.3 such that the final chosen mesh (denoted as Mesh-15) included 2978 vertices, 5802 faces and a mean edge length equal to 68.2 μm. The sub-study reported in Supplementary Material 2 demonstrated that such coarsening did not result in a loss of important geometrical features. Specifically, the coarsening induced error in geometry was randomly distributed over the bone surface, the mean error was 0.22 μm and the maximum error of 1.5 μm was located in a relatively flat area of the bone surface.

### 2.6 Mapping bone images using deformable image registration

Three-dimensional deformable image registration was used to individually map the binarized reference bone image to each of the remaining 21 (also binarized) individual bone images (Figure 1, Step 4). The Sheffield Image Registration Toolkit (ShIRT) was used for mapping, employing a “registration grid” with a “nodal spacing” (NS) of five voxels. Details of the algorithm can be found elsewhere (Barber and Hose, 2005). Briefly, ShIRT superimposes a cubic registration grid with a given NS on both the “fixed” (an individual bone) and the “moved” (reference to be mapped) images. It then determines the mapping between the two images as a displacement vector at each registration grid node. The displacement at any reference image voxel location or reference surface mesh vertex is computed by tri-linearly interpolating the displacements at its eight closest registration grid nodes. Adding the displacement of a reference surface mesh vertex to its position gives the position of an “anatomically similar” location on the individual bone surface.

Since the performance of the algorithm is sensitive to its spatial resolution (and hence NS) and the inherent bone surface differences in the input images, an evaluation study optimized the choice of the NS considering a combination of sources of complexities in the images. Similar to past studies (Dall’Ara et al., 2014), this was done by applying known “virtual” displacement fields to representative images, predicting this displacement using ShIRT, and quantifying the difference between known and predicted displacements for a range of NS values. Six studies, described below, define virtual displacement fields of increasing complexity by successively including new sources of uncertainty. The images of the six mice at 18 weeks in the “OVX + ML” group are representative of the full dataset. The known virtual displacements were used to synthetically generate six corresponding fixed bone images. ShIRT was used to register the fixed/moved image pairs with NS ranging from 5 to 50 voxels in steps of five voxels. The absolute difference between the imposed displacement and that estimated by ShIRT was calculated at bone boundary voxels, i.e., voxels whose voxel neighbors were not all bone or not all background. For a given NS, the average and standard deviation of the absolute differences, taken over all bone boundary voxels of all six moved images, are referred to as errors associated with the accuracy and precision of registration, respectively. As the execution time to register a typical pair of images was ∼15 min with NS = 5 voxels but ∼35 min with the smallest allowable NS = 2 voxels, NS lower than five voxels were not considered in the following. Similar evaluations, using the same algorithm for registering images of the same resolution and same bone structure, considered Nodal Spacings larger than 10 voxels (Dall’Ara et al., 2017). For all six studies, the bone images obtained after binarization and geometry correction in section 2.4 above were considered as the initial moving images..

**FIGURE 3:**
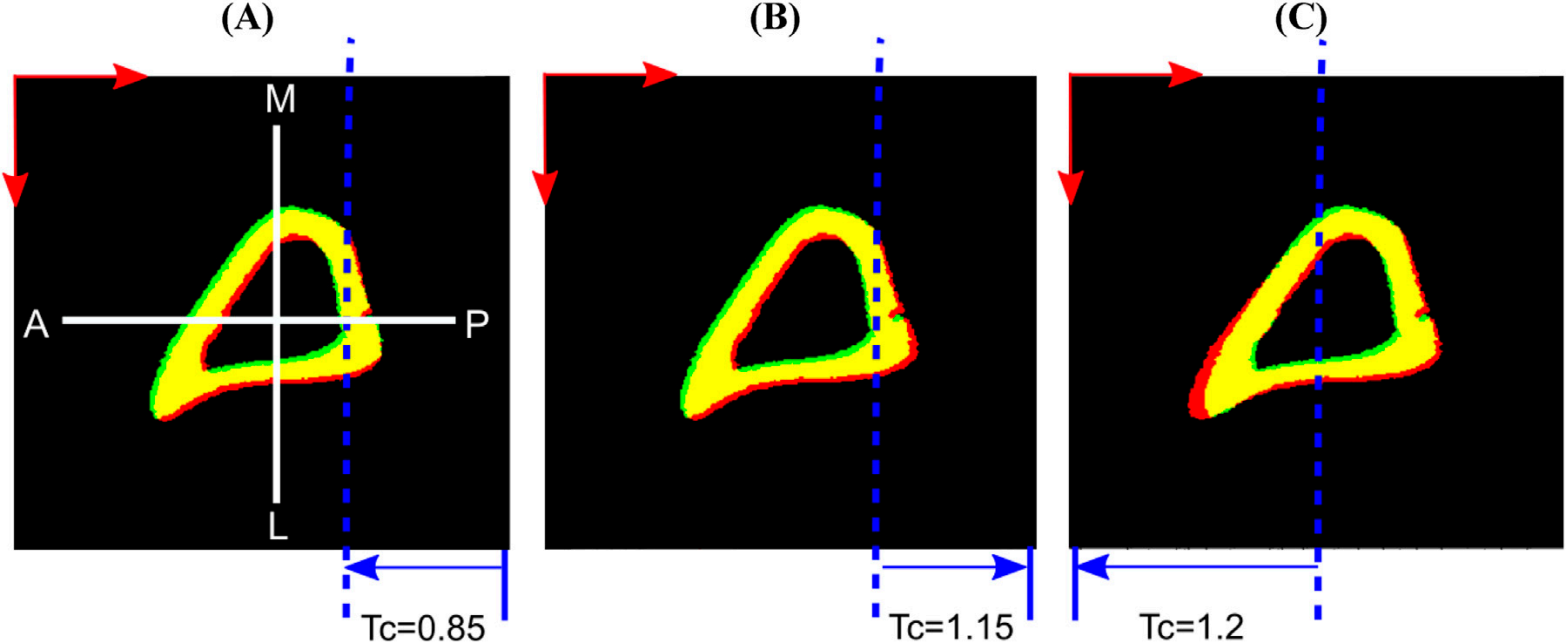
Examples of applied virtual deformation fields: **(A)** Study #5: Posterior, Smaller Part, Tc = 0.85; **(B)** Study #5: Posterior, Smaller Part, Tc = 1.15; **(C)** Study #5: Anterior, Half, Tc = 1.2. The deformations comprise: a virtual translation with components 2.5 voxels in horizontal (anterior, A to posterior, P) and vertical (medial, M to lateral, L) directions (red arrows), and two voxels in the length direction (proximal to distal, not shown here); and an affine transformation that deforms only the part of the image spanned by the blue arrows, displaces the dashed blue line by zero and reaches its full magnitude (Tc) at the corresponding edge of the image. Regions of overlap between the fixed (red) and moved (green) bone images are shown in yellow.

1. Uniform translation of binarized images: This study investigated the effects of rigid misalignments of the bone samples on the performance of the elastic registration. It tested the ideal scenario of uniform translations, equal to an integer number of voxel size. Displacements of either 2, 4 or 6 voxels each in the three Cartesian directions were individually applied to the moved images to obtain the fixed images.
2. Non-uniform translation on binarized images: Similar to Study #1, this study investigated the effects of the non-uniform rigid misalignments of the bone samples on the elastic registration. Thus, a displacement of 2 voxels along both the *x* and *y* directions and 4 voxels in the *z* direction was applied to obtain the fixed images.
3. Non-uniform, fractional voxel translation on binarized images: This study measured the effect of rigid registration, including interpolation errors due to the translations being equal to non-integer number of voxels. To test this, a translation of 2.5 voxels along both the *x* and *y* directions were first applied to the images. These were then resampled using bilinear interpolation and finally a translation of 2 voxels in the *z* direction was applied to obtain the fixed images.
4. Translation on greyscale images followed by binarization: The same translation as in study #3 was applied, but to the greyscale images of the six mice at 18 weeks in the “OVX + ML” group, as obtained before binarization and geometry correction steps in section 2.4. Binarization and geometry correction were applied to the translated images to obtain the fixed images. Study #4 is similar to Study #3, with the only difference being the binarization coming after the translation and interpolation. Therefore, this study includes the effect of applying the rigid registration firstly on grayscale images and also investigates how the binarization and geometry correction influences the deformable registration performance. This case is more representative of the order of the image processing steps as proposed in the current PCA framework.
5. Local deformation: This study evaluated the performance of the ShIRT algorithm to capture non-uniform and local deformations. The local deformations introduced by affine transformation were used to simulate the shape differences that exist between bone samples in the examined population. To do so, simulations of several local deformation fields were tested. Three affine transformations along the *x* direction were separately applied on the binarized images as obtained at the end of study #4. The three individual affine transformations were: (i) compression of the posterior half of the bone image by 0.95× (referred to as ‘Study #5: Posterior, Half, Tc = 0.95’), (ii) compression of a smaller posterior section of the image by 0.85×, 0.95× or stretching by 1.15× (referred as ‘Study #5: Posterior, Smaller Part, Tc = .’), and (iii) stretching the anterior half by 1.2× (referred as ‘Study #5: Anterior, Half, Tc = 1.2’). The third case was motivated by the observation that when any individual mouse bone image was overlaid on the reference image, the horizontal distance between the bone surfaces at the anterior crest was at most 11 voxels apart. The stretching factor of 1.2 achieved an imposed displacement of similar magnitude along the *x*-direction at the anterior crest. Figure 3 illustrates examples of virtual translation and affine transformation successively added to the original images, as evidenced by incomplete overlap between the obtained binarized deformed images (fixed) and the binarized original images (moved).
6. Image noise: Finally, this study examined the effect of image noise on the performance of the elastic registration. To do so, original image noise was measured from the original input image and subsequently added to the image to create a simulated noisy image. These steps were accomplished as follows. For each mouse in the “OVX + ML” group at 18 weeks, 3D image masks were created to separate the bone tissue and background regions in the grayscale images. The standard deviations of the pixel intensity in these regions were separately computed and then averaged over the six mice, leading to one standard deviation value each for bone and background. These were used to define two normal distributions (both with zero mean) from which samples were drawn and added to the bone and background regions of the greyscale mouse images to create simulated noisy images. The simulated images were then deformed as described in study #5. This study was carried out for NS equal to 5 and 10 voxels.

All virtual translations described above were performed using ImageJ 1.53s. Images were manipulated with affine transformation and noise in Matlab R2022b.

Once the coordinates of the “anatomically similar” locations of the discretized endosteal and periosteal surfaces were obtained for each bone shape observation, these were concatenated to construct a single “shape vector”

$$x_{j}=\left\{x_{j}^{1},y_{j}^{1},z_{j}^{1},x_{j}^{2},y_{j}^{2},z_{j}^{2},\ldots ,x_{j}^{N},y_{j}^{N},z_{j}^{N}\right\}$$

where $\left(x_{j}^{i},y_{j}^{i},z_{j}^{i}\right)$ are the Cartesian coordinates of the *i* ^th^ point (*i* = 1, 2, … , *N* = 2978) of the *j* ^th^ bone shape observation (*j* = 1, 2, … , *M* = 22). Here, *N* and *M* are the number of vertices in Mesh-15 and the number of tibia observations respectively. A data matrix ***P*** is constructed where the *j* ^th^ row constitutes the centered shape vector $x_{j}-x_{0}$ where $x_{0}$ is a (3*N*)-element row vector denoting the mean shape. The shape variance was calculated from the product of the ***P*** with its self-transpose. The sum of all the diagonal elements divided by the *N*−1 gives the total variance, whereas the sum of the diagonal elements corresponding to a cohort group or age gives the proportional variance of a specific subgroup within the examined population.

### 2.7 Decomposition into mode shapes and validation

The matrix ***P*** is decomposed using PCA as

P=a⋅Y

where a is the matrix of mode scores (dimension *M* × (*M*–1)) and Y is the matrix of mode vectors (dimension (*M*–1) × (3*N*)). The *M* scalar values in the *k* ^th^ column of the matrix a (*k* = 1, 2, … , *M*–1) represent the contributions to the *M* tibia observations due to the *k* ^th^ Principal Component (or mode). The shape of this mode is given by the *k* ^th^ row of the matrix Y .

Leave-one-out tests were conducted to assess the accuracy of reconstructing any tibia not belonging to the original database. In turn, the two shape observations (corresponding to weeks 18 and 24) of each of the six mice in the “OVX + ML” group, were randomly removed. PCA was performed on the remaining data, i.e., (5 “OVX” + 5 “OVX + ML”) × 2 ages. For each PCA mode, the score values of the two left-out sample tibiae were found by projecting these on the mode shape. The left-out samples were reconstructed as the sum of the mean shape and the linear superposition of the mode shapes, weighted by the score values. The reconstruction error was defined as the distance between corresponding surface nodes of the two pairs of reconstructed and left-out samples. Note that reconstruction errors were assessed only for the six pairs of shape observations in the “OVX + ML” group to evaluate the sensitivity of the PCA model to describe treatment-related variations over time.

### 2.8 Temporal variations and treatment effects categorization

**FIGURE 4:**
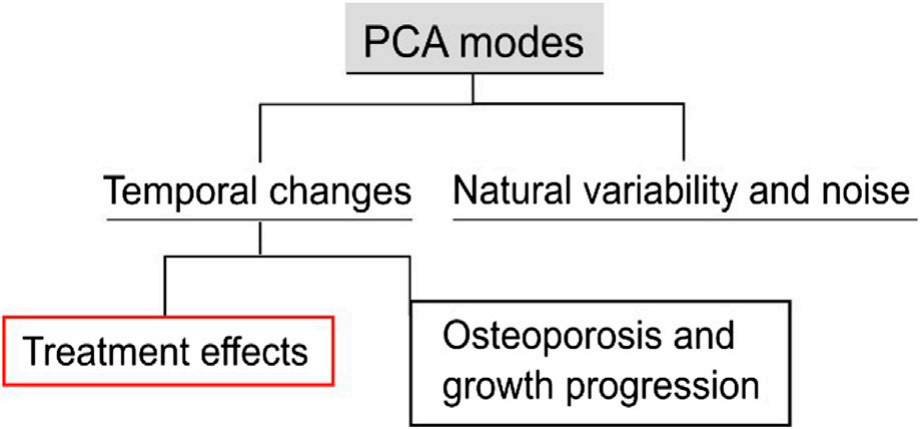
Types of variations in the examined population. The population includes longitudinal data of treated and untreated mice. The proposed PCA score processing uncovers all the sources of variations and differentiates them into classes. These classes are generic variations among groups and systematic variations with respect to time. The latter can be further categorized into disease progression and growth, and treatment effects.

In the current application, longitudinal data of treated and untreated mice were analyzed, i.e., 22 observations, to investigate the disease progression for the control group, “OVX”, and the treatment progression for the “OVX + ML” group. The 21 PCA modes describe multiple sources of variations. These variations can be categorized (Figure 4) as either a combination of natural variability between individual mice and random errors in the image processing framework or a temporal change. For modes that are not associated with temporal changes, it is expected that their scores will remain similar over time for an individual mouse. Therefore, the change over time in the scores of each mode was computed for all individual mice. Only those modes are considered as associated with temporal change for which median score changes over time are statistically significantly different from zero in either or both the “OVX + ML” and “OVX” groups. Two-sided Wilcoxon signed rank test was used to test if the temporal score changes in any group are significantly different from zero. Additionally, in order to evaluate whether the “OVX + ML” and “OVX” at week 18 are biologically indistinguishable, Wilcoxon signed rank test on the scores of these subgroups was performed. A nonparametric test was used because of the small data sample size.

Temporal changes were further distinguished into either an effect of treatment or a combined effect of other temporal factors, e.g., ovariectomy and growth. Here, three possibilities arise: 1) effect only due to treatment: evidenced by a non-zero median score change in only the “OVX + ML” group; 2) effect not due to treatment: evidenced by a non-zero median score chfange in only the “OVX” group, 3) effect partially due to treatment: evidenced by non-zero median score changes in both “OVX” and “OVX + ML” groups. The Mann–Whitney U test was performed to compare the two cohorts with each other. Where a mode is established to be associated with a temporal change, this change was quantified in two ways. First, Cohen’s *d* effect size was computed as the ratio of the average and standard deviation of the score changes in the group (Lakens, 2013). Second, the surface change that each mode describes was computed by considering the median score changes of the “OVX + ML” group between two ages and scaling the mode vectors. Specifically, a centered shape vector $P_{j18}^{k}$ , of the j^th^ shape observation at week 18, can be reconstructed using a treatment-related mode $Y^{k}$ , *k* = 1, … , *M*–1 using the PCA formula. Similarly for the j^th^ shape observation at week 24. Therefore, the two reconstructions of the j^th^ shape observation of a group at the two ages can be written as

$$P_{j18}^{k}=a_{j18}^{k}\cdot Y^{k}$$

$$P_{j24}^{k}=a_{j24}^{k}\cdot Y^{k}$$

The surface change SC of the j^th^ shape reconstruction using the k^th^ model is the difference between the coordinates of $P_{j24}^{k}$ and $P_{j18}^{k}$ , and it can be written as:

$${SC}_{j}^{k}=P_{j24}^{k}-P_{j18}^{k}=\left(a_{j24}^{k}-a_{j18}^{k}\right)\cdot Y^{k}$$

To represent a group, the median of the surface change is:

$${MSC}^{k}=median\left[{SC}_{j}^{k}\right]=median\left[\left(a_{j24}^{k}-a_{j18}^{k}\right)\right]\cdot Y^{k}$$

The median surface change of a group is henceforth called surface change for the sake of brevity. Note that the surface change has dimensions of length. The direction of the vectors indicates bone formation or bone deletion as geometric changes on the active surfaces.

## 3 Results

### 3.1 Mapping bone images using deformable image registration

In study #1, the registration algorithm ShIRT determined the known displacement of uniform rigid translation by an integer number of voxels, leading to a maximum error (across the range of NS investigated) of 2.5 × 10^−5^ voxels (2.6 × 10^−4^ μm) associated with accuracy and of 1.3 × 10^−4^ voxels (1.3 × 10^−3^ μm) associated with precision. In study #2, where a non-uniform rigid translation (2 voxels in *x* and *y* directions and four voxels in *z*) was applied, the registration errors were similarly negligible, as a maximum error associated with accuracy of 2.8 × 10^−5^ voxels (2.9 × 10^−4^ μm) and associated with precision of 1.7 × 10^−4^ voxels (1.8 × 10^−3^ μm) were obtained.

**FIGURE 5:**
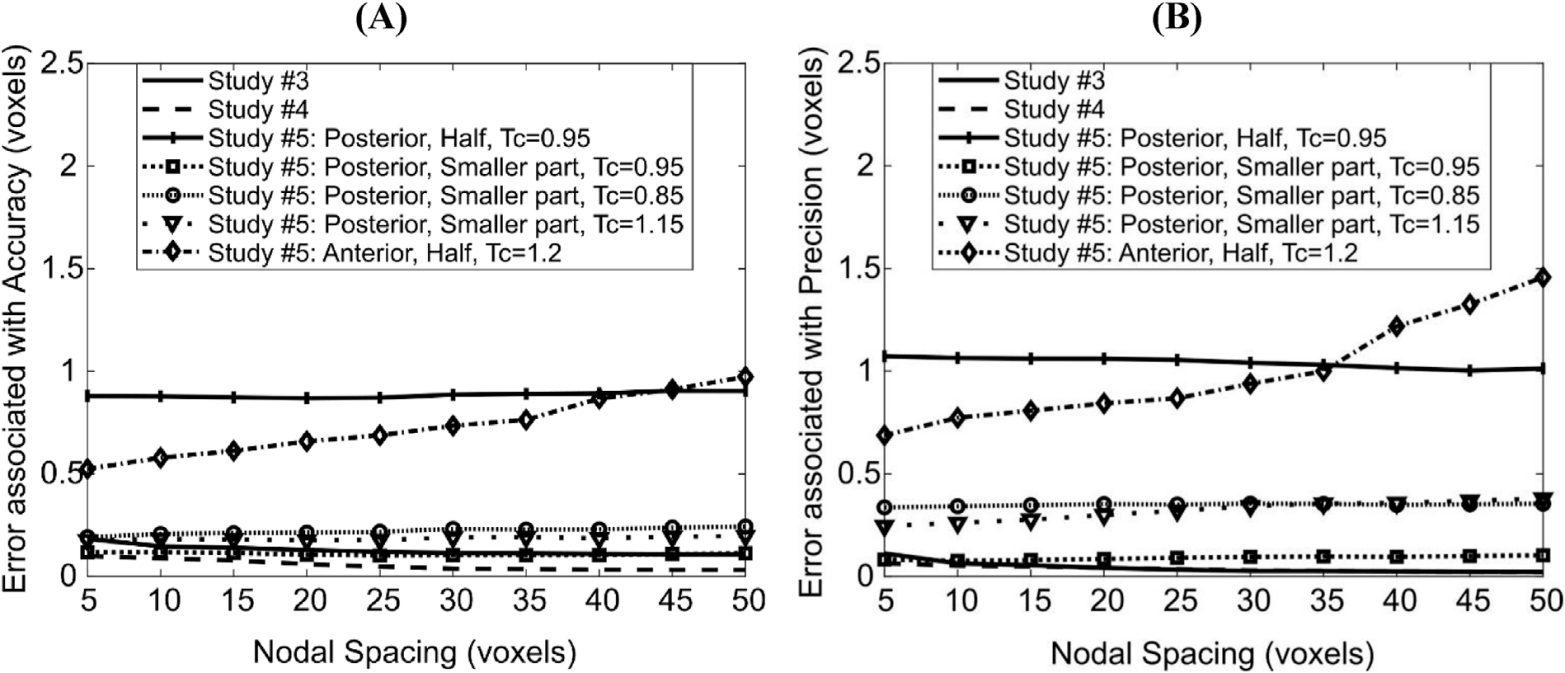
Dependence of errors associated with accuracy **(A)** and precision **(B)** of the deformable registration on Nodal Spacing. Each line corresponds to a different imposed displacement field; the details of these are found in the main text.

In study #3, rigid translation by a non-integer number of voxels led to constant errors (across the range of NS investigated) of ∼0.15 voxels (1.56 μm) in magnitude associated with accuracy (Figure 5A) and ∼0.05 voxels (0.52 μm) in precision (Figure 5B). Note that translation with a non-integer number of voxels involved blurry transition due to the linear interpolation of black and white pixels and then forced back to binary values (thresholding). This possibly explains the higher error magnitudes in this study compared with those in studies #one to two.

On the other hand, the same translation field on grayscale images revealed lower errors associated with accuracy (∼0.08 voxels = 0.83 μm, Figure 5). Note that in study #4 linear interpolation is performed on a spatially smooth field of grey values, which results in a smoother final bone surface than in study #3, which probably explains the lower errors associated with accuracy. The errors associated with precision were similar to those in study #3. The slight decrease in errors associated with accuracy and precision with large NS in both studies was because the output displacement fields were smoother, possibly reducing a noise effect and getting closer to the uniform imposed virtual translation.

The displacements applied in ‘Study #5: Posterior, Half, Tc = 0.95’ led to almost constant errors of ∼0.87 voxels (9 μm) associated with accuracy and ∼1.1 voxels (11 μm) associated with precision. The displacements applied in ‘Study #5: Posterior, Smaller Part, Tc = …’, led to errors associated with accuracy of ∼0.20 voxels (2.3 μm) for affine transformation coefficient Tc = 0.85, ∼0.12 voxels (1.2 μm) for Tc = 0.95 and ∼0.18 voxels (1.8 μm) for Tc = 1.15. Errors associated with precision were nearly constant around 0.34, 0.08 and 0.26 voxels (3.5 μm, 0.8 μm, 2.7 μm, respectively) when Tc = 0.85, 0.95 and 1.15 respectively. Comparing ‘Study #5: Posterior, Half, Tc = 0.95’ and ‘Study #5: Posterior, Smaller Part, Tc = 0.95’ suggests that errors associated with both accuracy and precision increase as a larger part of the image is deformed. Comparing within ‘Study #5: Posterior, Smaller Part, Tc = …’ suggests that the errors associated with accuracy and precision are higher when the magnitude of the difference between the affine transformation coefficient and unity increases (Tc = 0.85/1.15 vs. 0.95, i.e., |Tc–1| = 0.15 vs. 0.05). It also revealed that the errors increase when the direction of the deformation and of rigid transformation oppose each other (shrinkage: 0.85, Figure 3A) instead of being aligned (expansion: 1.15, Figure 3B).

**FIGURE 6:**
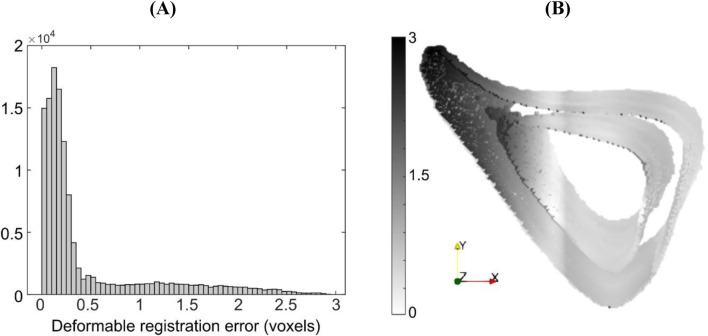
**(A)** Histogram of registration errors and **(B)** their spatial distribution on the bone surface for the simulated displacement field given by ‘Study #5, Anterior, Half, Tc = 1.2’. Errors are shown for a representative specimen taken from the “OVX + ML” group at 18 weeks of age. Contour darkness indicates error magnitude at the specific location on the bone surface.

For the case ‘Study #5: Anterior, Half, Tc = 1.2’, the errors associated with accuracy varied between 0.52 and 0.97 voxels (5.4–10 μm), whilst those associated with precision varied between 0.68 and 1.45 voxels (7.1–15 μm). Both types of errors increased with an increase in NS, in a monotonic fashion. The higher errors in precision are due to the localization of large magnitude errors around the anterior crest, which is expected because the imposed displacement magnitude is highest in that region (Figure 6A). For NS = 5, the maximum error at the anterior crest was 2.8 voxels (29 μm), which is less than 25% of the maximum imposed simulated local deformation (12 voxels = 124 μm). The 75% (interquartile range, IQR = Q3–Q1) of the surface locations were successfully registered with a systematic error smaller than 0.47 voxels. The median registration error over the bone surface and for all mice was 0.18 voxels (1.8 μm) (Figure 6B).

The addition of image noise (Study #6) led to 0.56 voxels (5.8 μm) and 0.73 voxels (7.6 μm) in errors associated with accuracy and precision respectively for NS = 5, averaged across all mice, and to 0.59 voxels (6.1 μm) and 0.78 voxels (8.1 μm) in errors associated with accuracy and precision respectively for NS = 10. For NS = 5, the median registration error over the bone surface was 0.33 voxels (3.4 μm), and its spatial distribution was similar to when image noise was absent for the same nodal spacing with slightly higher standard deviation (‘Study #5: Anterior: Half, Tc = 1.2’).

After this step, the mesh samples were consistently discretized, with a fixed number of nodes. The shape variance as described by the centered mesh data in the groups “OVX” week 24 and “OVX + ML” week 24 amounted to 18% and 44% respectively of the total variation of geometry in the data set. The remaining variation in the dataset was due to all mice at week 18.

### 3.2 Decomposition into mode shapes and validation

**FIGURE 7:**
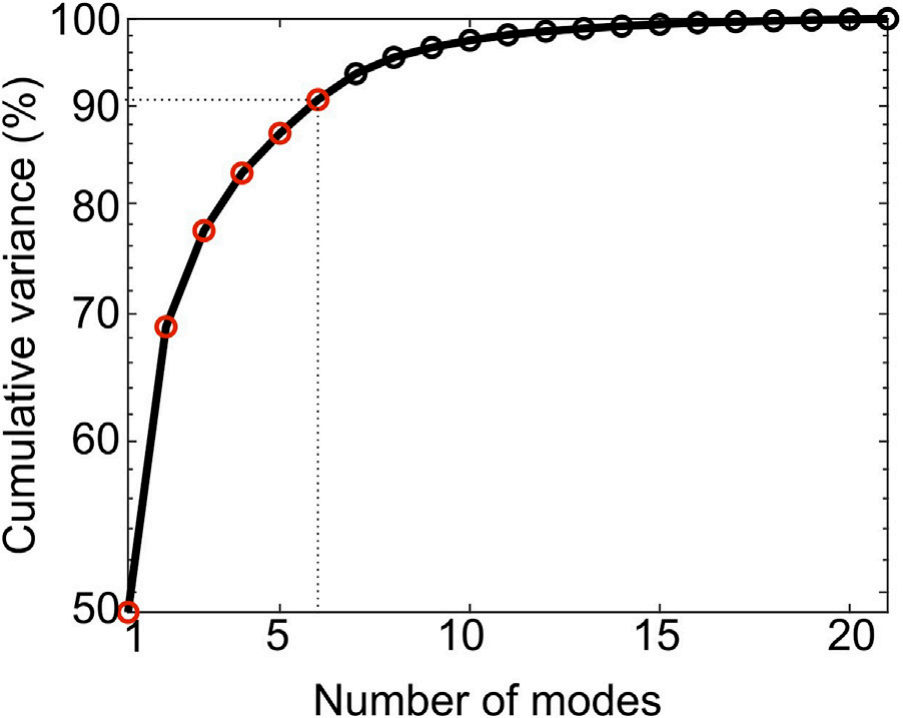
Cumulative variance (%) explained by the PCA modes. The first six modes (red circles) describe up to 91% of the total geometric variance within the examined population.

The first 6 PCA modes explained 91% of the total variation in tibia geometry (Figure 7). All remaining modes (7–21 modes) individually explained 2% or less of the total variation in tibia geometry.

**FIGURE 8:**
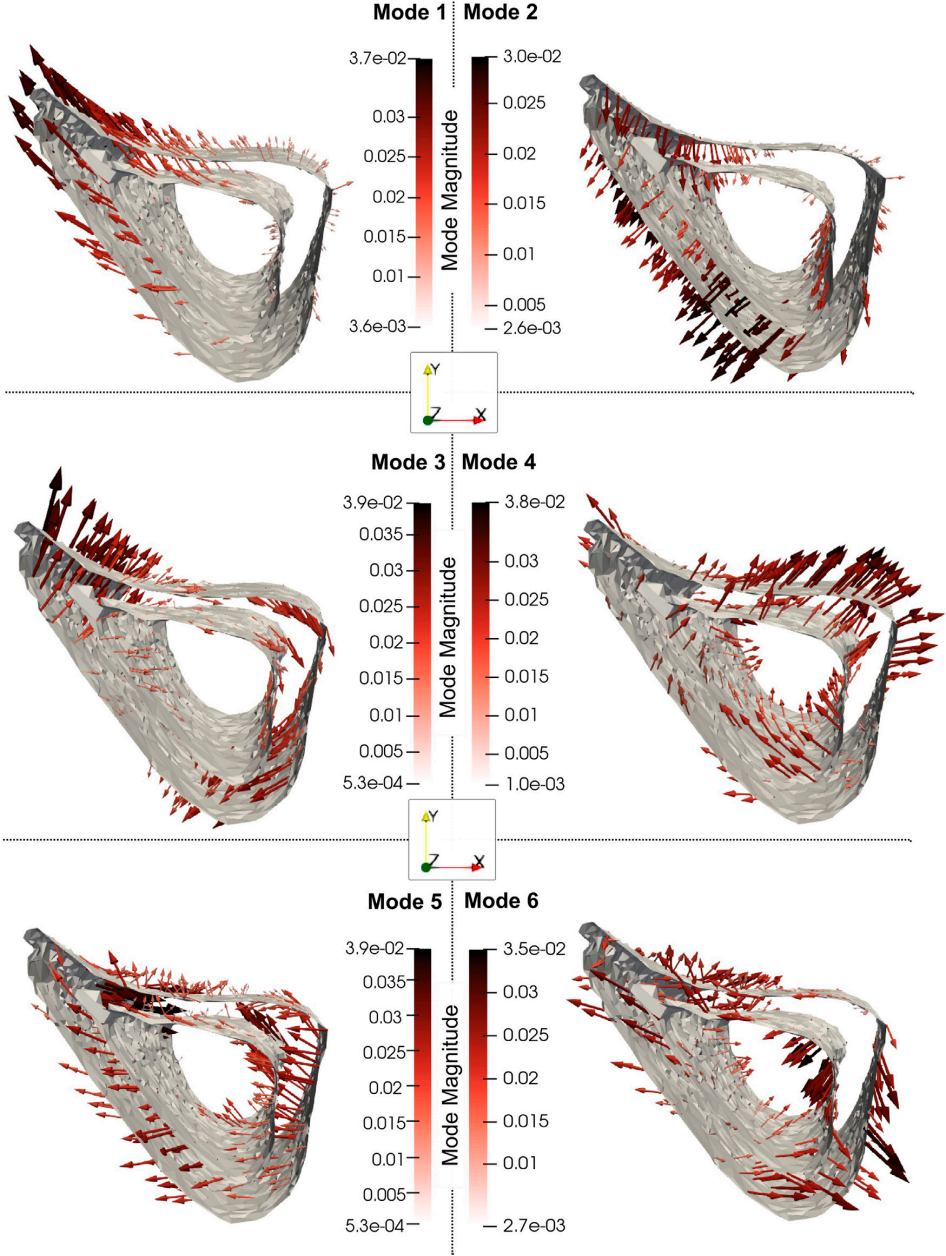
The 3D profiles of the treatment-related modes 1, 2, 3, 4, 5 and 6, depicted as vectors plotted on the mean shape. The vectors are scaled and colored by the mode magnitude at each point of the mesh. Darker and longer arrows indicate higher variability in shape across different bone specimens at that surface location. All profiles share the same viewpoint.

As shown in Figure 8, Mode 1 (explaining 49% of total shape variation) describes variations in the thickness and “sharpness” of the anterior crest and Mode 2 (explaining 20%) describes variations at the endosteum at the medial aspect. Mode 3 (explaining 8%) describes variations in the curvature of the medial and lateral aspects and Mode 4 (explaining 6%) describes local variations at the lateral aspect of the distal end. Figure 8 also shows that Mode 5 (explaining 5%) describes local variations at the distal anterior crest and Mode 6 (explaining 3%) describes variations in local features scattered all over the bone midshaft, of which the variations at the posterior periosteal and posterior-lateral endosteal surfaces are prominent.

**FIGURE 9:**
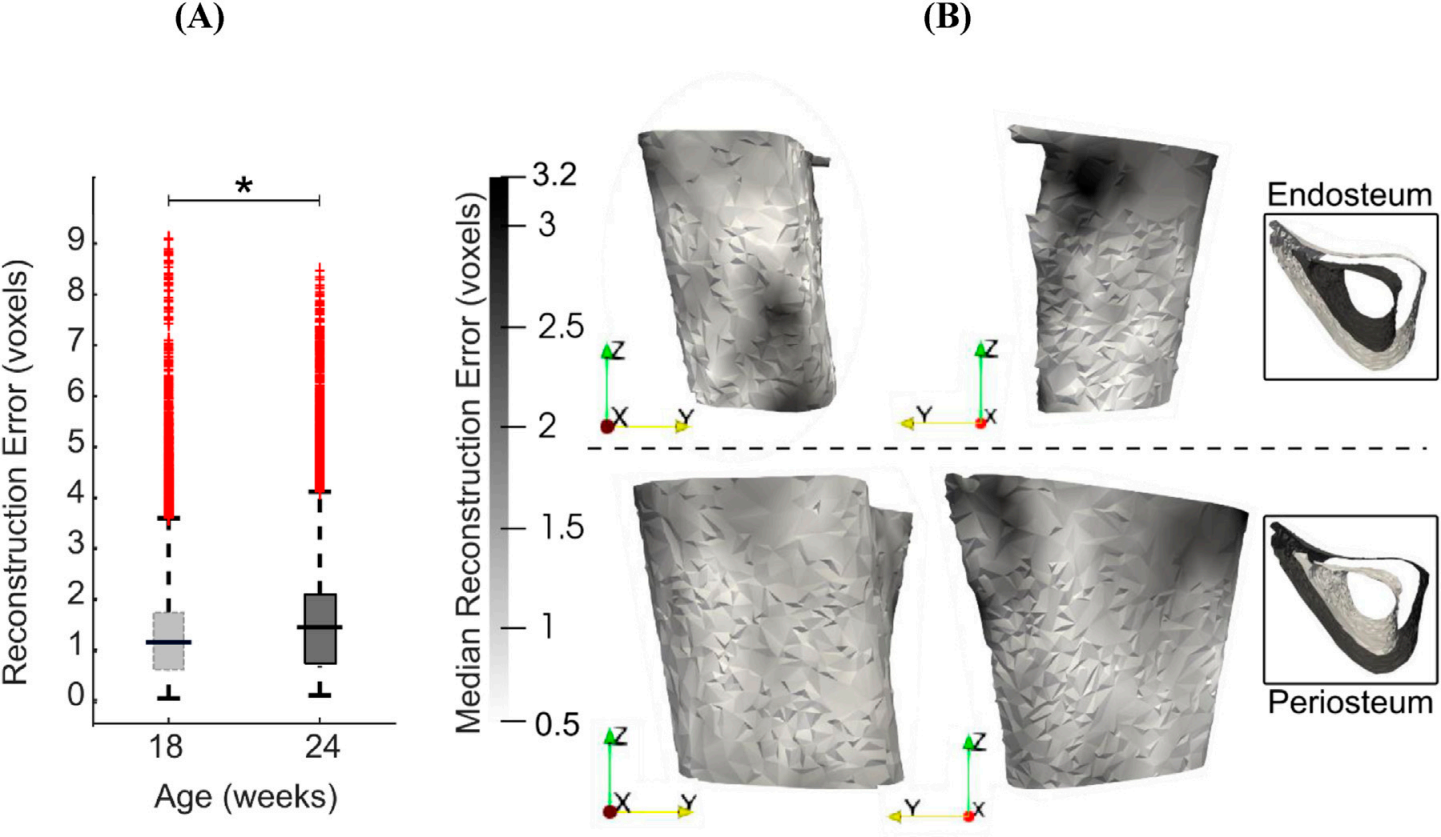
**(A)** Boxplots of the leave-one-out errors in reconstructing the bone geometry of six mice in the “OVX + ML” group at weeks 18 and 24. The overall median error for week 24 is statistically significantly higher (p < 0.05) than for week 18. **(B)** Two different views of the endosteal and periosteal surfaces (mean bone shape for week 24) overlaid with contours levels indicating magnitude of median error at each bone surface location for week 24.

Reconstruction errors in the leave-one-out tests had an average, standard deviation and maximum of 1.5, 1.2 and 9.5 voxels (16 μm, 12 μm and 99 μm) respectively when considered over all surface points and all mouse samples. Figure 9 shows the boxplots of the error distribution over the tibia surface for specimens grouped by age. The large number of outliers in both distributions highlights their skewness. The median errors in week 18 (1.2 voxels, 12 μm) were lower than those in week 24 (1.4 voxels, 15 μm) (Figure 9A, *p* < 0.05). Figure 9B shows the median error distribution on the 3D bone profile over the six mice at 24 weeks of age. Relatively higher error magnitudes are found around the proximal medio-posterior and anterior edges and at the distal latero-posterior aspect in the endosteum only, but errors are otherwise small and randomly distributed over the bone surface.

### 3.3 Temporal variations and treatment effects categorization

**FIGURE 10:**
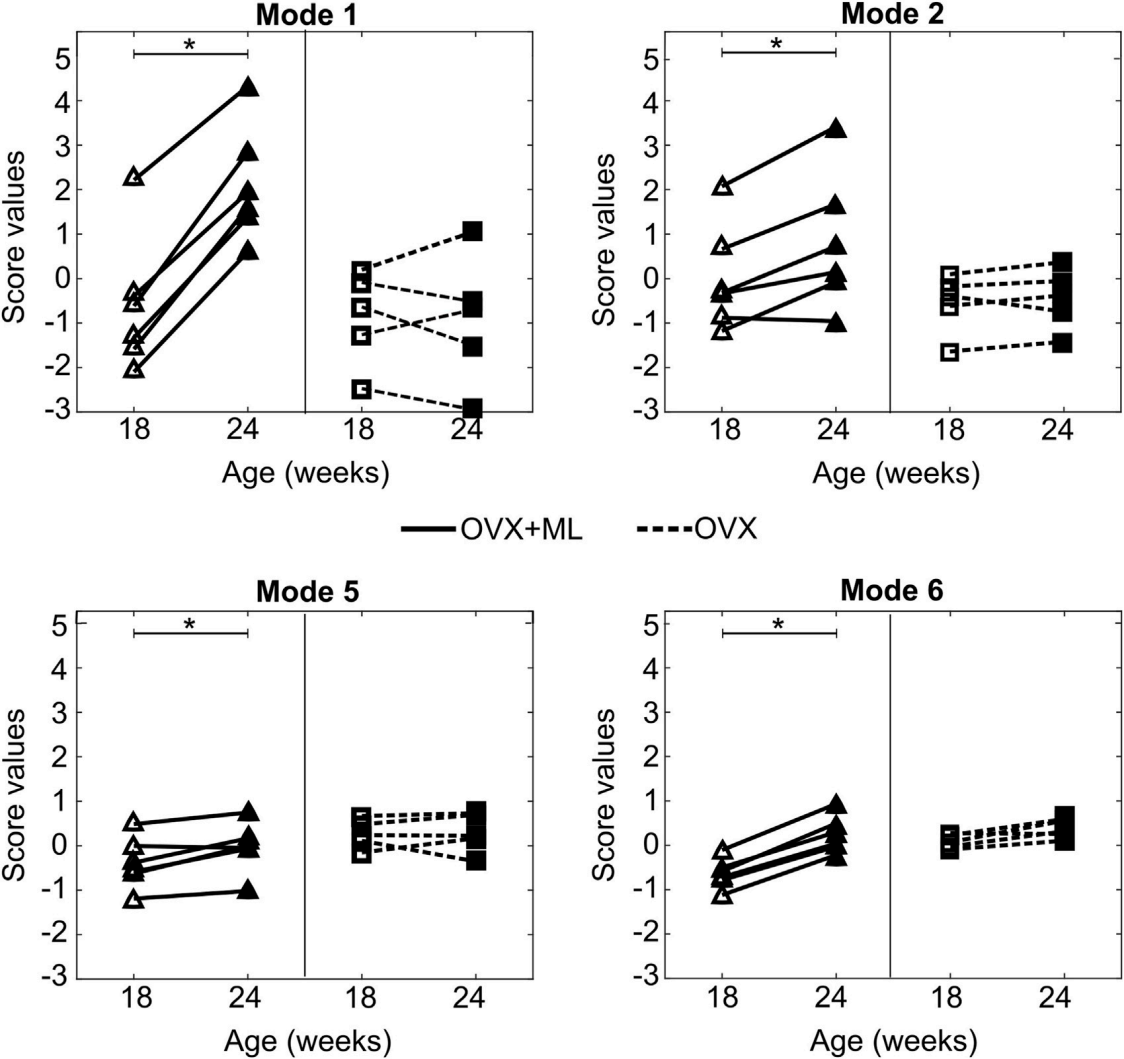
Treatment categorization of Modes 1, 2, 5 and 6. Mode score values of individual mice at are shown at week 18 (unfilled) and week 24 (filled) (“OVX + ML”, △; “OVX”, ▢). Lines (“OVX + ML”, solid; “OVX”, dashed) connect mode scores of individual mice between the two time points. Asterisks (*) highlight median changes with time of mode scores in a group that are statistically significantly (p < 0.05) different from zero.

Mouse-specific changes in mode scores, going from week 18–24 of age, were statistically significantly different from zero only for Modes 1, 2, 5 and 6 in the “OVX + ML” (*p* < 0.05) group (Figure 10). Additionally, no statistically significant differences (*p* > 0.05) between “OVX + ML” and “OVX” groups at week 18 were found when comparing their scores of these PCA modes. As such, Modes 1, 2, 5 and 6 describe geometric features that are associated with a temporal change, and this change is an effect only due to treatment. This could be also visually indicated by the positive trend of score changes for the “OVX + ML” group in Figure 10, in contrast with score changes for the “OVX” group which do not follow a specific pattern. Modes 1, 2, 5 and 6 were found to have Cohen’s *d* effect sizes of 2.0, 0.60, 0.54 and 2.4 respectively.

**FIGURE 11:**
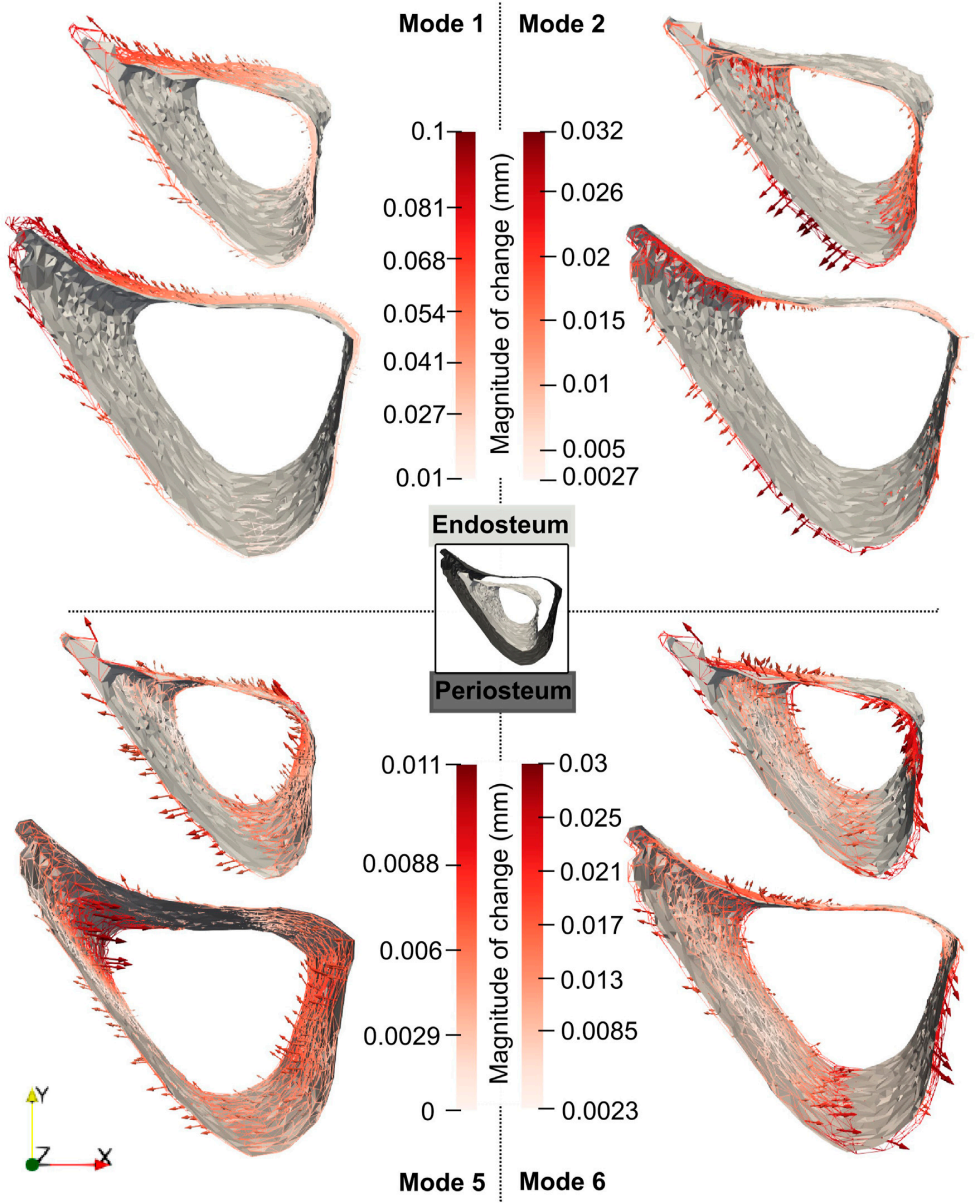
Median changes in endosteum and periosteum shapes due to Modes 1, 2, 5 and 6 between weeks 18 and 24 in “OVX + ML” group. The directions of change at different locations are denoted by the arrows, and a redder arrow indicates a relatively larger change. The mean bone profile at week 18 is shown as a solid gray surface, whilst the mean profile at week 24 is given as a colored wireframe.

For these modes, the profiles of the mode-specific temporal surface changes are shown in Figure 11. This figure illustrates the change map on top of the median mode-specific bone profile at week 18. The arrows and magnitudes show that Mode 1 is associated with prominent endosteal deletion of 0.054 mm and periosteal formation of 0.1 mm at the anterior crest along the whole length of the midshaft and less prominent concurrent endosteum and periosteal formation (0.027 mm) at the latero-posterior aspect. A similar interpretation revealed that Mode 2 describes endosteum deletion and periosteal formation in the range of 0.013–0.032 mm along the medial side. Mode 5 primarily captured periosteal deletion at the distal end of the anterior crest with a magnitude of 0.011 mm. Although this opposed the effect described by Mode 1, the summative effect of both Mode 1 and Mode 5 is still periosteal expansion of the anterior crest by 0.09 mm. Finally, Mode 6 exhibited endosteal formation at the posterior-lateral aspect of 0.03 mm and periosteal formation along the posterior side of similar magnitudes (0.03 mm).

## 4 Discussion

The main objectives of this study are to demonstrate that: (a) the variations in mouse bone geometry obtained by the novel framework are robust to uncertainty sources contained therein; (b) to demonstrate that this framework enables an assessment of 3D geometric variations induced by treatment.

### 4.1 Robustness of framework including mapping bone images

The robustness of the framework was assessed through case studies focusing on its distinct steps. These analyses were conducted for all mice in the “OVX + ML” group at the age of 18 weeks. At this age, the groups “OVX + ML” and “OVX” were shown to be biologically indistinguishable (*p* > 0.05, statistical tests on the scores) as the mechanical loading interventions start only when mice are 19 weeks old.

All images were rigidly registered to a reference structure at the starting time point (Viceconti and Dall’Ara, 2019), to eliminate spatial errors (Campbell et al., 2014). Since the comparison of the longitudinal images is very sensitive to the registration of each set of microCT scans, a validated registration procedure with errors of less than 3.5% was used (Lu et al., 2016), demonstrating reproducibility in similar mouse tibia studies (Oliviero et al., 2022). Finally, as this study focused on one segment within the entire tibia volume, further local registration at the tibia midshaft was successfully implemented aided by its simple geometry (compared with more heterogeneous trabecular regions).

The first evaluation study (Supplementary Material 1) demonstrated that cortical pores and trabeculae occupy a very small portion of the total bone section volume, and that excluding these features had negligible influence on the mechanical strain distribution. As such, this simplification was considered acceptable. It is not a limitation of the framework because the state-of-the-art method of standard morphometric analysis at midshaft also excludes such features. The second evaluation (Supplementary Material 2) indicated that bone surface topology was preserved across various levels of discretization, with errors converging quickly and remaining small compared to voxel size.

Since ShIRT is here used for a new application for mapping bones from different mice and ages, the third evaluation study (section 3.1) assessed the accuracy and precision of the algorithm in the prediction of uniform, non-uniform, global and local surface displacements. Mean errors in mapping such surfaces were always below one voxel. SD of errors were almost always lower than one voxel, apart from local and high magnitude deformations which seemed to challenge the algorithm. In those cases (‘Study #5: Anterior, Half, Tc = 1.2’), a linear relationship between errors and NS was revealed and errors associated with precision were found higher than one voxel for NS > 35 voxels. Since the distribution of errors in the bone surface was skewed resulting in high errors in the most deformed bone areas, NS was chosen to be five voxels to ensure that all important local differences within the examined population of shapes are captured. For NS = 5, errors remained smaller than one voxel (5.8 μm and 7.6 μm errors associated with accuracy and precision, respectively) in the presence of existing and simulated image noise and when binarization and geometry correction steps were also applied. Taken together, this evaluation study suggests that the mapping of the reference bone surface to “anatomically similar” locations on the 21 mouse tibial surfaces, achieved using deformable registration with NS = 5 voxels, contains errors associated with accuracy and precision smaller than one voxel (10.4 μm).

Past evaluation studies of ShIRT have found comparable error magnitudes and similar relationships between error magnitudes and NS of deformable registration. For example, Dall’Ara et al. (2014) showed that when predicting uniform displacements of two voxels using NS = 5 voxels, the mean and standard deviation of error magnitudes were of the order of 10^–4^ voxels and 10^–2^ voxels respectively. Dall’Ara et al. (2017) simulated uniform virtual displacement fields of similar magnitude in murine tibia samples and found good performance in precision. In the present evaluation study #1, where uniform or non-uniform virtual displacements (i.e., homogeneous but anisotropic) of integer magnitudes (up to six voxels) was used, mean and standard deviation of error magnitudes were of the order of 10^–5^ voxels and 10^–4^ voxels respectively, and relatively independent of NS, and similar to those reported in the two studies mentioned above. However, whilst the cortical midshaft section of murine tibia was analyzed in the present study, Dall’Ara et al. (2014) focused on localized cubes extracted from trabecular or cortical regions of bovine femur. The lower error magnitudes found in the present study are expected because the topology of the cortical region of the murine midshaft is much simpler than of the trabecular bovine femur regions.

A non-uniform translation equal to a non-integer number of voxels results in significantly larger mean errors, as also demonstrated previously (Dall’Ara et al., 2014). It is attributable to interpolation, but with less impact on grayscale images. This is expected because interpolation of the displacement on the bone surface is more gradual when applied to grayscale images. Local deformations led to non-uniform errors on the bone surface, with error magnitudes being larger in areas of larger local deformation (Figure 6). Therefore, the dependence of the errors on NS is modulated by the deformed features and the magnitude of deformation. For example, a flatter shape of the posterior part in ‘Study #5: Posterior, Half, Tc = 0.95’ resulted in a relatively low dependence of error magnitudes on NS, similarly with applying local deformation in a small fraction of the image. In contrast, the sharp feature of the anterior part explains the linear increase of errors with NS in the case of ‘Study #5: Anterior, Half, Tc = 1.2’. Image noise slightly increased registration errors. In this study, artificial noise was added to already noisy images, while Dall’Ara et al. (2014) used noise from repeated scans. Despite the difference in noise sources, error magnitudes were similar to those reported previously. The strong dependence of registration errors on NS for complex simulated displacement fields suggests the need to reassess this dependence for new applications. Such reassessment should ideally design tests representative of the length scale, image resolution, alignment, features and noise that are present in such new applications.

Simulated displacement fields, such as those described in the present evaluation study, have the advantage that different sources of complexity can be separately analyzed. However, not all past PCA models have analyzed registrations errors in similar ways, which makes it challenging to directly compare the magnitude of errors reported here with earlier work. Unlike the present study, where images were mapped using elastic registration, Bryan et al. (2010) registered the surface meshes. The defined registration error as the distance between the registered and target surface meshes were reported with mean and maximum errors of 0.60 mm and 3 mm, respectively. Their error magnitudes cannot be directly compared (i.e., in dimensional units) to the error magnitudes reported in the present study, due to differences in bone sites, scales and imaging modalities. As the image resolution in the study of Bryan et al. (2010) was 0.78 mm×0.78 mm×2 mm, the errors can be inferred to be in the range of 0.3–3.8 voxels. A similar range of mean registration errors (0.42–3 voxels) was reported by Brown et al. (2014) and Brown et al. (2017) who imaged hind limbs of female C57BL/6 (similar bone size as the present study) with somewhat lower resolution (14 µm). Registration errors in the present study are slightly smaller than those reported in the above studies. This could be due to differences in elastic registration approach between current and past work and errors in initial positioning/alignment that existed in these past studies, but these effects cannot be further separated.

### 4.2 Decomposition of variations and treatment effects categorization

Among all groups, the “OVX + ML” week 24 group contributed most to the total variation. This indicates that it is furthest from the mean shape of the whole dataset and suggests a strong effect of treatment. The relatively small variation within the “OVX” week 24 group indicates that the effect of untreated ovariectomy from the period from 18 to 24 weeks cannot be reliably distinguished from the natural variation between mice in this study. The results of standard morphometric analysis on the same mice suggest that the variation in cortical area (both week 18: 19%; “OVX” week 24: 13%, “OVX + ML” week 24: 68%) and in cortical thickness (both week 18: 18%; “OVX” week 24: 11%, “OVX + ML” week 24: 71%) are distributed similarly (Roberts et al., 2019; Roberts et al., 2020). However, while the novel framework presented here can be used to assess the variation in the full 3D bone geometry, standard morphometric analysis can be used to assess only the part of variation in geometry that is captured by the morphometric parameters.

The geometry variation across the 22 mouse image specimens was compact, with 90% of it being explained by the variation of only six principal components (i.e., shape modes). This is interesting because several different factors, including natural variability between individuals, growth, disease, treatment and artifacts of image processing were present in the dataset. The full 3D assessment enabled by PCA allows a concise description of separate findings of previous studies. Mode 1 describes simultaneous formation and deletion localized at the anterior crest, which agrees with the previous findings of Cheong et al. (2020b). At this site, the mode vectors on the formation surface are longer than those on the deletion resorption surface indicating higher magnitude of change in periosteum than endosteum (0.1 mm vs. 0.054 mm), and this agrees with previous findings of Birkhold et al. (2017). This localized cortical thickening of 0.046 mm also agrees with the averaged cortical thickening of 0.064 mm of similar bone region reported by Roberts et al. (2020). Roberts et al. (2020) attributed this change to treatment, and this agrees with the present finding that Mode 1 is characterized as a pure treatment effect (by the statistical tests of the scores). An *a posteriori* analysis found that Mode 1 was moderately correlated (*R* ^2^ > 0.50) to the standard morphometric parameters (extracted from the same dataset): maximum moment of inertia (*R* ^2^ = 0.62), minimum moment of inertia (*R* ^2^ = 0.55) and eccentricity (*R* ^2^ = 0.61) and had a low correlation with area and thickness. Correlations between any of the Mode 2–6 scores and any morphometric parameter (area, thickness, maximum and minimum moment of inertia and eccentricity) were consistently low (*R* ^2^ ≤ 0.50) or negligible.

Mode 2 describes periosteal formation and endosteal deletion of the medial aspect which could potentially explain the increased cortical area in the study of Roberts et al. (2020). The localized changes in the anterior–medial aspect agree with the predictions of Razi et al. (2015) and Javaheri et al. (2020) that indicate higher strain magnitudes at these locations. Both Mode 1 and 6 also describe localized thickening of the posterior-lateral edge, consistent with other loading murine studies which highlight the dependency of the bone remodeling to the strain distribution (Carriero et al., 2018; Cheong et al., 2021; Sugiyama et al., 2008; Rooney et al., 2023). Overall, posterior and anterior bone response as shown by Modes 1 and 6 are also in agreement with the local thickness changes as measured cross-sectionally in the midshaft slice of the tibia bone in similar loading models (Miller et al., 2021; Holguin et al., 2014). The periosteal deletion of the distal anterior crest as described by Mode 5 has not been previously reported elsewhere but this effect is much smaller compared to the opposite effect of Mode 1. Overall, the current framework provides a full 3D assessment of the geometric changes, that could not be obtained by previous approaches.

The used sample size (n = 6 for each examined cohort and age) was relatively small but similar to other longitudinal studies that quantify bone changes over time and space (Li et al., 2019; Lu et al., 2017; Roberts et al., 2019). The overall median reconstruction errors in the “OVX + ML” groups in weeks 18 and 24 were comparable to the standard deviation of cortical thickness (0.5 voxels and 1.7 voxels respectively) as reported previously by Roberts et al. (2019, 2020) using the same images. This implies that the difference in cortical thickness between an original and a reconstructed geometry (using all PCA modes) of a mouse is of the same order of magnitude as the difference in cortical thickness between two randomly selected mice in the population. This suggests that a mouse not belonging to the examined population cannot be reconstructed using the discovered PCA modes sufficiently accurately. This highlights that inferences drawn based on the somewhat small dataset must be extrapolated with caution to a larger population of mice undergoing the same course of disease/treatment.

Notwithstanding this limitation, some assertions regarding modes corresponding to treatment effects can still be made reliably. The median reconstruction error across all “OVX + ML” week 24 samples is the highest (∼3 voxels) at the anterior crest. The median change due to Mode 1 is also highest (∼9 voxels) at a similar location. This suggests that the small dataset used in the present study does not limit the assertion that Mode 1 is an effect of mechanical loading treatment. A similar assertion can be made for Mode 6, as the median change corresponding to this mode is the highest at the endosteum (∼3 voxels) which is nearly twice the highest median reconstruction error (found at a similar anatomical position). However, such assertions cannot be made for either Mode 2 or Mode 5, as the maximum median change (3 and 1 voxels, respectively) is not distinguishable from the magnitude of median reconstruction errors in the medial aspect and distal anterior crest, respectively.

The higher confidence in interpreting Modes 1 and 6 as treatment effects is supported by their large effect sizes. The contrast in fractions of total variation explained between Modes 1 and 6 highlights that Mode 1 describes a global change (a large area around the anterior crest) whereas Mode 6 describes a local change (a small area on the posterior-lateral aspect). Modes 2 and 5 explain larger fractions of total variance than Mode 6 but approximately a quarter of the effect size of Mode 6. This highlights that Modes 2 and 5 do not capture treatment effects as reliably as Mode 6, even if these modes separately explain larger fractions of the total variance than Mode 6.

The orthogonality of PCA modes guarantees that the treatment effects described by Modes 1, 2, 5 and 6 are uncorrelated to each other. In contrast, the standardized set of morphometric parameters, such as cortical thickness and area, are dependent on each other. This makes it challenging to separate the fractions of total variation explained by each parameter. In the present study, the treatment-related modes described most of the total variance, indicating the role treatment plays in modifying bone shape. Orthogonality of modes also allows attributing Modes 3, 4, 7–21 to sources of variation other than treatment, such as natural variability including random errors and noise but not growth and disease which have systematic temporal effects. The lack of a temporal effect was supported by our analysis of the original data in Roberts et al. (2019). This analysis showed that neither cortical thickness or cortical area were statistically significantly different (Wilcoxon signed-rank test, *p* > 0.05) between 18 and 24 weeks in the “OVX” group. This is expected because the impact of ovariectomy in murine bone models is prominent in the first couple of weeks post-surgery but stabilizes after that period (Roberts et al., 2019).

A major advancement due to the proposed framework is that the experimental design (and corresponding imaging data) drives the discovery of geometric features that automatically separate into independent effects due to treatment, unpacked from disease, growth, random variations and combinations of these, whilst capturing the full 3D variation in the data. This contrasts with the state-of-the-art approach of fixing the morphometric parameters to characterize bone geometries. The study in this paper successfully demonstrated that there are important local treatment effects that the scalar parameters cannot describe. This contrasts with prevailing approaches that discard a part of total shape variation and moderately decompose the effects of treatment, disease, growth and/or random variations along these parameters, with no guarantee that the decomposition is mutually independent.

### 4.3 Limitations

The 90% coarsening of the surface meshes showed that important geometrical features across the reference bone section are preserved (Supplementary Material 2). Similar levels of coarsening were also suggested previously in the study of Brown et al. (2017). However, a concern is whether the coarse reference surface mesh is representative of all bone samples across the tibia length. Specifically, if there are important features that are present in different regions of bone surfaces for other bones, then coarsening the reference surface mesh cannot guarantee that these features are described by the mapped reference meshes following deformable registration. In the present study, the size of the cropped section of the tibia was proportional to its length, which ensured that these sections corresponded to similar anatomical regions and thus reduced the possibility of including disparate anatomical landmarks.

This framework is designed to investigate bone variations within an examined population. Although this is based on a PCA model, the latter should be used with care when attempting to approximate new geometries not observed in this study. It is also important to note that individual PCA mode shapes and scores, even those with the highest explanatory power or effect size (Modes 1 and 6), cannot predict the full effect of treatment for individual mice, and should not be used in a predictive modeling setting. Therefore, the number of samples used in this study are insufficient for the purpose of building *in silico* physiologic or pathologic cohorts. However, this study can contribute to an ongoing process of data sharing within the scientific community with the long-term aim of reducing the use of animals in bone research (Viceconti and Dall’Ara, 2019).

This study assumed negligible densitometric variations within the bone samples. Roberts et al. (2020) found 1% change in the tissue mineral density (TMD) in a similar bone segment driven by a double course of mechanical loading. Oliviero et al. (2021) analyzed homogeneous (Young’s modulus *E* = 14.8 GPa) and heterogenous (*E* derived from local TMD) FE models of mouse tibia at different ages and cohorts (healthy, diseased and treated) but did not find any significant differences in bone mechanics. In future applications where TMD is expected to change significantly (above levels reported in Roberts et al. (2020)) or be more heterogeneous compared to (Oliviero et al., 2021), further development of the present framework is necessary to describe local TMD variations and separate it into effects of treatment, disease, growth, natural variability and other relevant sources.

This application only focused on the midshaft section, and further development is needed to analyze 3D shape variation in the entire tibia. It is expected that removing the fibula (Cheong et al., 2020b) will be necessary to preserve the topological similarity. The high dimensionality of the problem and the computational demand of each step of the framework should be carefully considered. Here, controlling the surface mesh density using coarsening could be a crucial step. Sophisticated job submission techniques may be needed for efficient compute time and data management. The application of conventional PCA in the framework presented here inherently assumes that modes can be linearly combined. This assumption might occlude important effects, and the use of several alternative models, such as Probabilistic PCA (Kim and Lee, 2003) and Gaussian processes (Lawrence and Hyvärinen, 2005) should be explored in the future.

The presented PCA-based framework is limited to the analysis of topologically equivalent shapes. As such, only the variations in cortical bone regions can be examined and the framework excludes the possibility of analyzing trabecular regions. The image processing protocol for removing cortical pores and trabeculae was based on previous evidence of bone remodeling for this specific bone system. However, if the framework is applied to a different volume, site of bone or different bone, evidence of bone remodeling and influence on bone mechanics should be reviewed in the new context.

The selected mouse genetic strain was considered skeletally mature at week 14 of their age and an appropriate experimental model to quantify bone variations in osteoporosis and treatment cohorts. However, bone aging can affect bone response to interventions. Ageing-related effects were not distinguished in this analysis. Particularly, the PCA database did not include any healthy specimens, but it considered ovariectomized mice as the control group. Additionally, some sources of uncertainties within the examined population that could not be eliminated are the exact date of birth related to their age (± some days), genetic phenotypes, success of ovariectomy (complete removal of ovaries with/without additional soft tissue) and recovery.

## 5 Conclusion

The proposed new framework based on longitudinal microCT imaging, image processing and PCA on discretized bone shapes showed the potential to improve the preclinical treatment investigation in murine bone models. The framework was demonstrated to accurately describe bone shapes up to ∼1 voxel accuracy in the presence of several error sources in the processing pipeline. Application of PCA on discretized tibial midshaft shapes taken from a population of diseased and treated mice identified for the first time six mutually independent geometrical features that explained a significant fraction of the total variation in the 3D imaging data. Four of these geometric features (modes) were found to be purely an effect of the mechanical loading treatment and described changes over the course of treatment at the anterior crest, medial aspect, posterior area and some specific localized features. Due to the small dataset size, only two of these features could be reliably asserted as being treatment effects. Nevertheless, these features offer a compact description of several changes found in previous studies, and contain new information not discovered until now. The imaging data used here enabled the demonstration of the various methodological aspects of the developed framework, which was the primary focus of this paper. However, the application of the framework is not limited to the experimental set-up from where the images are sourced. It has the potential to be used as a more precise strategy for investigating the effect of different treatment strategies on bone structure.

## Data Availability

The raw data supporting the conclusions of this article will be made available by the authors, without undue reservation.
