# Supplementary material for: A novel framework for elucidating the effect of mechanical loading on the geometry of ovariectomized mouse tibiae using principal component analysis
Source: Front Bioeng Biotechnol. 2024 Oct 22;12:1469272. doi: 10.3389/fbioe.2024.1469272 (PMC11534826; doi:10.3389/fbioe.2024.1469272)
Supplement: Supplementary file 1 [file DataSheet1.docx]

***Supplementary Material 1: Mechanical importance of cortical pores and trabeculae in the midshaft section***

## Methods

The second step of the proposed framework, i.e. binarization and geometry correction (Section 2.4), ensures the topological equivalence of the shape observations to an annular cylinder. This step locally “corrected” the geometry, such as by removing trabeculae and cortical pores. The aim of this study is to quantify the influence of these local geometry corrections on the mechanical response.

Two micro-Finite Element (microFE) models of each midshaft tibia section were created by directly converting all bone voxels from the 3D images (original and processed) into linear hexahedral elements (Cheong et al., 2020). The ratio of integration points (IPs), present in either model but not both, to the total number of the IPs in the original model was computed as a measure (denoted M1) of difference in overall volume due to geometry correction. For both models, homogeneous isotropic material properties were assumed, $E=14.8$ GPa, $\nu= 0.3$ (Oliviero et al., 2018). All nodes on the top surface were constrained to have the same displacement as a control node to which a $1$ N static load was applied in the axial direction. All translation degrees of freedom of all nodes on the bottom surface were fixed. The above microFE modelling pipeline has been previously validated against experimental measurement of stiffness (errors of 14% ± 11%) and failure load (errors of 9% ± 9%) (Oliviero et al., 2018). MicroFE analyses were performed using Abaqus 2018. At the IPs common to both models, the absolute difference in minimum principal strain was computed, normalized by the maximum absolute minimum principal strain in the original model and denoted as measure M2*.* This measure indicated the overall difference in mechanical strain between the models. For both models, “highly compressed” IPs were identified as those with the 10% smallest minimum principal strain (Oliviero et al., 2021). Of these, only those IPs that are common to both models were counted, the ratio of this to the total number of the highly compressed IPs in the original model was determined and denoted as measure M3. This measure indicates the degree to which the critically compressed region remains unchanged after geometry correction. For the highly compressed IPs common to both models, the absolute difference in minimum principal strain was determined, normalized by the maximum absolute minimum principal strain in the original model and denoted as measure M4. This measure describes the effect of feature removal on the failure load prediction. The area around the corrected features (trabeculae/pores) were identified as follows. Subtracting the 3D processed image from the original yielded the volumes of the removed trabeculae. Subtracting the original image from the processed images yields the volumes of cortical pores (filled). Image dilation by using a disk of 3 voxels was used to expand the volumes of these features, so that the analysis includes the surrounding area around the feature. The connectivity function was used to separate the features within the same bone geometry. The minimum and maximum *x*, *y, z* coordinates were determined for each feature separately. This defined the 3D bounding boxes that enclosed areas around each corrected feature. The number of the highly compressed IPs falling inside the bounding boxes enclosing filled cortical pores were counted. The ratio of this to the total number of highly compressed IPs (measure M5-cortical pores) indicates the portion of the fracture locations that is close to this specific type of corrected feature. M5-trabeculae was obtained similarly by considering bounding boxes enclosing deleted trabeculae. In the case of multiple features, the fraction with the highest value is retained.

## Results

The values of each measure, with mean and the standard deviation taken over six mouse samples of “OVX+ML” group at 18 weeks, are summarized in the table below. For measures M2 and M4, the average was taken over all relevant integration points and all mice. For measure M5, the mean value comes from the one specimen that exhibited a trabecula and as such no standard deviation can be computed.

*Supplementary Table 1: Statistics of measures of difference and similarity between original and geometry corrected model. Explanation of measures (M1, M2, …) are given in the main text.*

| *Metric* | *Mean* | *Std* |
| --- | --- | --- |
| *M1* | 0.40% | 0.15% |
| *M2* | 0.23% | 0.82% |
| *M3* | 95% | 2.0% |
| *M4* | 1.4% | 0.36% |
| *M5-cortical pores* | 0.5% | 0.94% |
| *M5-trabeculae* | 6.6🞨10^-3^% | – |

All measures are indicative of differences between the two models except M3 is a measure of similarity. Mean values of all difference measures are very low (<1.4%) and the mean of the similarity measure M3 is very high (95%). The mean values of measures M1–4 suggest that the effect of geometry correction on overall volume, overall strain, highly compressed volumes and critical strain values is negligible. The mean values of M5 measures suggest that the highly compressed volumes have negligible overlap with the region surrounding either type of corrected feature (cortical pore and trabecula). The standard deviations of all measures are very low (<2%) suggesting that the above inferences can be reliably asserted for all mouse samples. In conclusion, geometry correction has a negligible impact on predicting the global and local mechanical response of the bone section.

# Supplementary Material 2: Role of mesh coarsening on surface geometry description

## Methods

For each mouse, fifteen successively coarse surface meshes were obtained by reducing the number of faces by 20% at each step. Table 1 shows how the number of faces (actual output) and mean edge length depend on the desired number of faces (input) for the reference mouse bone. The coarsest mesh (Mesh-15) was created by 96% reduction of the faces in the original mesh and comprised 5802 faces with a mean edge length of 0.068 mm.

Two distance metrics were defined to quantify the effect of coarsening on shape representation accuracy, these are referred to as the maximum normal distance and the mean normal distance. For the reference mouse, $d_{ij}^{v}$ is defined as the normal distance between vertex $v$ of Mesh-i and its closest face on Mesh-j (i,j=0…15). The maximum and mean forward normal distances between Mesh-i and Mesh-0 are, respectively, the maximum and mean of $d_{i0}^{v}$ taken over all vertices $v$ of Mesh-i. The maximum and mean backward normal distances between Mesh-0 and Mesh-i are, respectively, the maximum and mean of $d_{0i}^{v}$ taken over all vertices $v$ of Mesh-0. Between a pair of meshes, the maximum normal distance is the larger of the maximum forward and maximum backward normal distances, and the mean normal distance is the larger of the mean forward and mean backward normal distances. This approach was used to evaluate both distance metrics for each mouse in the ‘OVX+ML’ group at 18 weeks old at different levels of surface coarseness. A level of surface coarseness was considered admissible if both distance metrics were below a threshold of 1 voxel ($0.0104 mm$) for all six mice. All levels of coarsening (up to 15) were found admissible according to the above criterion. Therefore, the coarsest mesh was chosen to reduce the computational demand of the subsequent steps of the framework.

*Supplementary Table 2: Number of faces and mean edge length for each coarseness level*

| *Coarseness step i* | *Number of faces* | *Mean edge length (*$\boldsymbol{mm}$*)* |
| --- | --- | --- |
| 0 | 166639 | 0.013 |
| 1 | 133181 | 0.015 |
| 2 | 106542 | 0.017 |
| 3 | 85230 | 0.019 |
| 4 | 68174 | 0.021 |
| 5 | 54537 | 0.024 |
| 6 | 43587 | 0.027 |
| 7 | 34821 | 0.030 |
| 8 | 27834 | 0.033 |
| 9 | 22250 | 0.037 |
| 10 | 17779 | 0.041 |
| 11 | 14208 | 0.045 |
| 12 | 11358 | 0.050 |
| 13 | 9080 | 0.056 |
| 14 | 7259 | 0.062 |
| 15 | 5802 | 0.068 |

## Results

The mean normal distance was found to rapidly increase in the first coarsening steps, but it gradually flattened after 7 coarseness levels (Supplementary Figure 1). After 13 coarseness levels the mean errors were only slightly increased to 1%. This trend revealed that the simplification algorithm converges into a certain level of surface description error and subsequently no coarser meshes were further tested. At any coarsening level, the mean and the maximum normal distance were smaller than the voxel size and did not exceed 0.22$\mu m$ and 1.5$\mu m$ for any mice (Supplementary Figure 2). Therefore, all coarsening levels met the admissibility criterion.

| **(A)** | **(B)** |
| --- | --- |
| 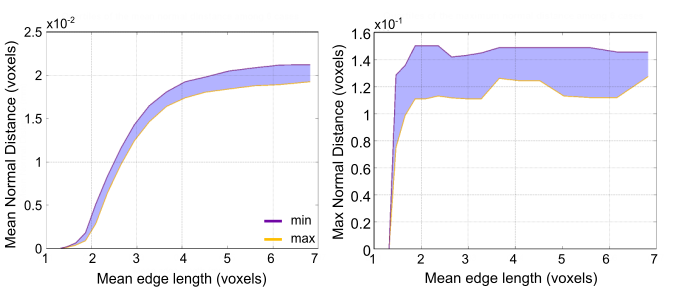 | 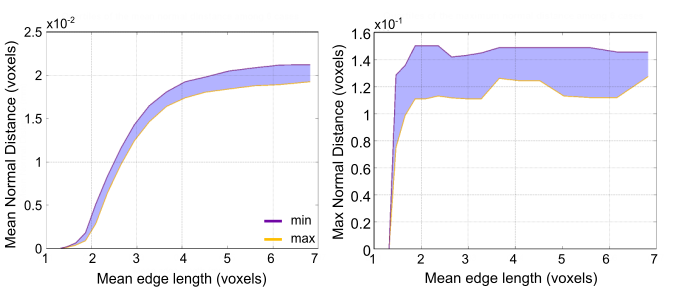 |

*Supplementary Figure 1: Dependence of surface discretization errors on the mean edge length derived from the sequential coarseness levels (A) Mean Normal Distance and (B) Maximum Normal Distance. Quartiles defined by the minimum and maximum values among 6 mouse sample are shown by solid purple and orange lines in both plots.*

| **(A)** | **(B)** |
| --- | --- |
| *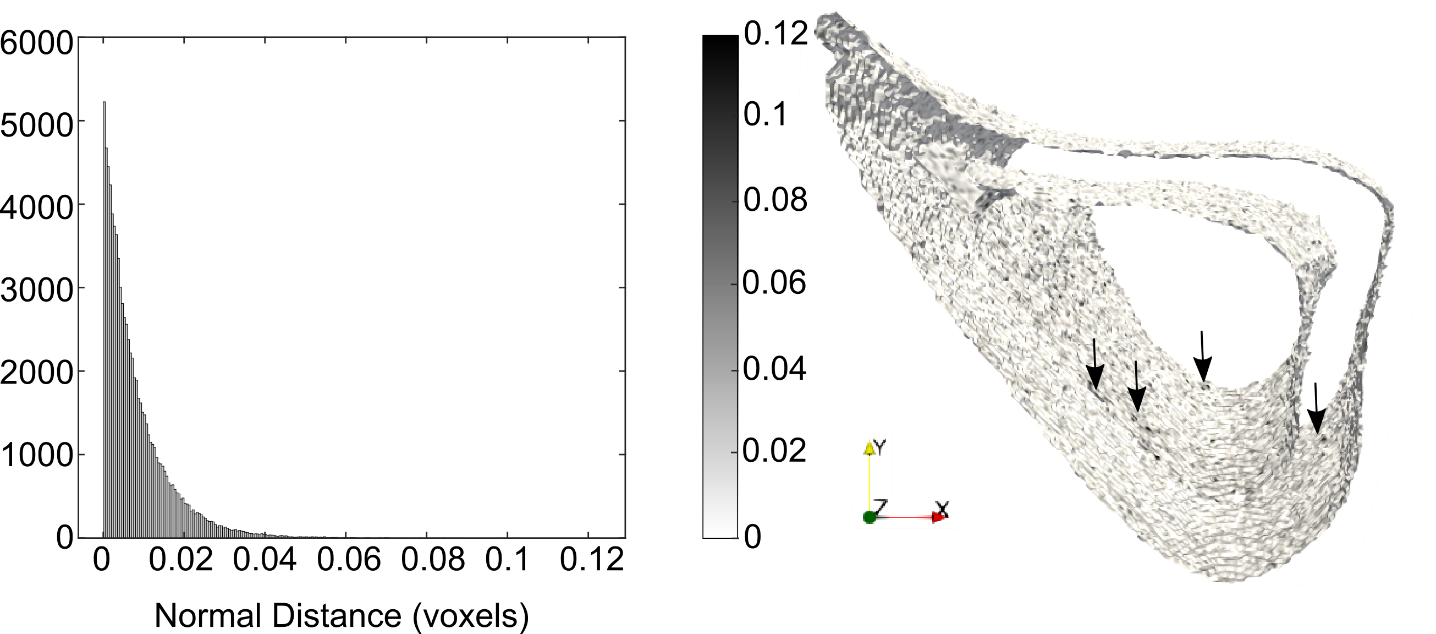* | *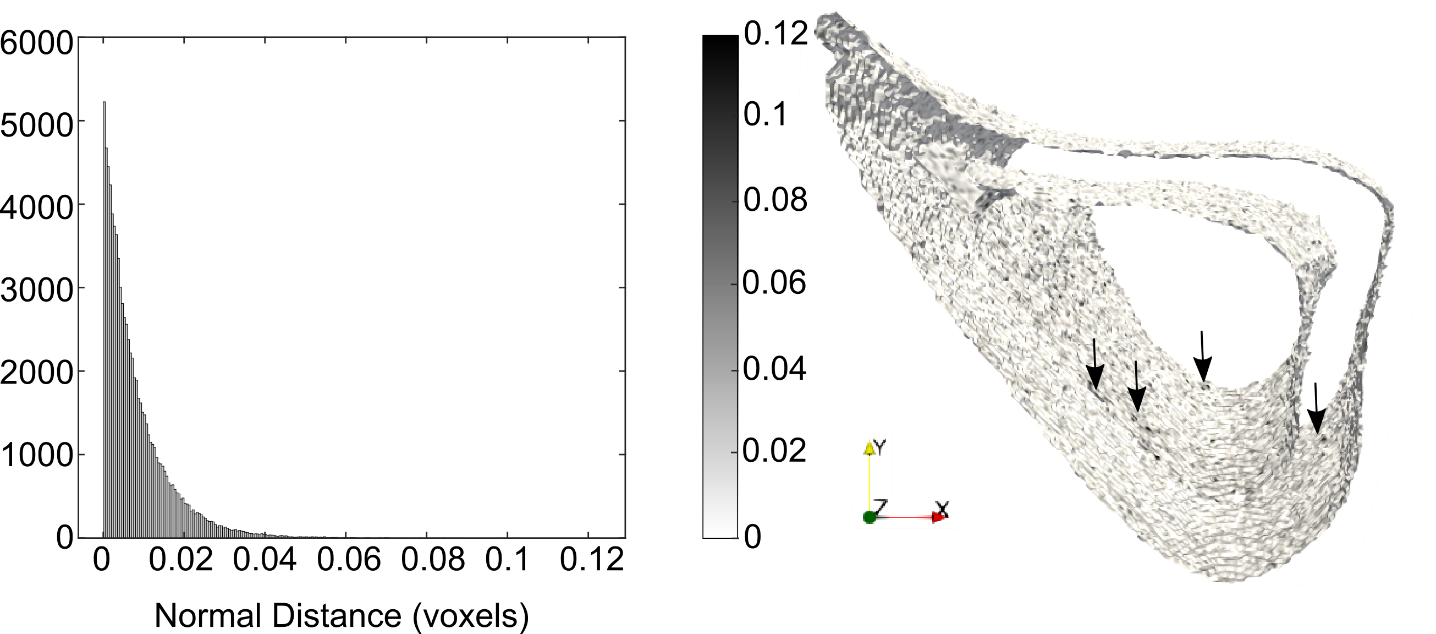* |

*Supplementary Figure 2: (A) Histogram of the surface discretization errors and (B) their spatial distribution on the bone surfaces. The error profile illustrates the forward normal distance from the finest to the coarsest mesh (Mesh-0 to Mesh-15). This histogram and profile describe only one specimen in the “OVX+ML” group and 18 weeks of age and is representative of other samples. The contour colors give the error magnitude and correspond to specific locations on the 3D shape. Black arrows highlight some areas with the largest errors.*

# References

Cheong, V. S., Campos Marin, A., Lacroix, D., & Dall’Ara, E. (2020). A novel algorithm to predict bone changes in the mouse tibia properties under physiological conditions. *Biomechanics and Modeling in Mechanobiology*, *19*, 985–1001. https://doi.org/10.1007/s10237-019-01266-7

Oliviero, S., Giorgi, M., & Dall’Ara, E. (2018). Validation of finite element models of the mouse tibia using digital volume correlation. *Journal of the Mechanical Behavior of Biomedical Materials*, *86*, 172–184. https://doi.org/10.1016/j.jmbbm.2018.06.022

Oliviero, S., Owen, R., Reilly, G. C., Bellantuono, I., & Dall’Ara, E. (2021). Optimization of the failure criterion in micro-Finite Element models of the mouse tibia for the non-invasive prediction of its failure load in preclinical applications. *Journal of the Mechanical Behavior of Biomedical Materials*, *113*, 104190. https://doi.org/10.1016/j.jmbbm.2020.104190
